# Effective cataract surgical coverage in adults aged 50 years and older: estimates from population-based surveys in 55 countries

**DOI:** 10.1016/S2214-109X(22)00419-3

**Published:** 2022-10-11

**Authors:** Ian McCormick, Robert Butcher, Jennifer R Evans, Islay Z Mactaggart, Hans Limburg, Emma Jolley, Yuddha D Sapkota, Joseph Enyegue Oye, Sailesh Kumar Mishra, Andrew Bastawrous, João M Furtado, Anagha Joshi, Baixiang Xiao, Thulasiraj D Ravilla, Rupert R A Bourne, Alarcos Cieza, Stuart Keel, Matthew J Burton, Jacqueline Ramke, the RAAB International Co-Author Group, Rosario Barrenechea, Maria Eugenia Nano, Anthea M Burnett, Nor T Lepcha, Levi Kandeke, Min Wu, Biaxiang Xiao, Beatriz Natividad Rodríguez Rodríguez, Juan F Batlle, Felipe A Chiriboga, Heba AlSawahli, Astrid V Villalobos, Jafer K Ababora, Robert P Finger, Manfred Mörchen, Mariano Yee Melgar, Doris M Alvarado, Xiu Juan Zhang, János Németh, Elesh Jain, Sucheta Kulkarni, Ahmad Ashraf Amalius, Lutfah Rif’ati, Mansyur Syumarti, Seyed Farzad Mohammadi, M Mansur Rabiu, Jefitha Karimurio, Joseph W Wachira, Hery Harimanitra Andriamanjato, Khumbo Kalua, Mohamad Aziz Salowi, Ubeydulla Thoufeeq, Pedro A Gomez-Bastar, C Van Lansingh, Sarah Polack, Ala Paduca, Uranchimeg Davaatseren, Reeta Gurung, Ram P Kandel, Sailesh Kumar Mishra, Yuddha D Sapkota, Nasiru Muhammad, Muhammad Zahid Jadoon, Nicholas Sargent, Ileana Brea, Alexander Páez, Rainald Duerksen, César Gonzales, Cristina I Eusebio, Wanjiku Mathenge, Saad Hajar, George E Kabona, Paddy B Musana, Grace C Mutati

**Affiliations:** International Centre for Eye Health; Clinical Research Department; International Centre for Eye Health; London School of Hygiene & Tropical Medicine, London, UK; Centre for Public Health, https://ror.org/00hswnk62" Queen’s University, Belfast, UK; International Centre for Eye Health; Grootebroek, Netherlands; Sightsavers, Haywards Heath, West Sussex, UK; International Agency for the Prevention of Blindness South-East Asia, Kathmandu, Nepal; Sightsavers Cameroon Country Office, Yaoundé, Cameroon; Nepal Netra Jyoti Sangh, Tripureswor, Kathmandu, Nepal; International Centre for Eye Health; Division of Ophthalmology, Ribeirão Preto Medical School, University of São Paulo, Ribeirão Preto, Brazil; Melbourne, VIC, Australia; Affiliated Eye Hospital of Nanchang University, Nanchang City, China; Lions Aravind Institute of Community Ophthalmology, Aravind Eye Care System, Madurai, India; Vision and Eye Research Institute, School of Medicine, Anglia Ruskin University, Cambridge, UK; Department of Noncommunicable Disease, World Health Organization, Geneva, Switzerland; Department of Noncommunicable Disease, World Health Organization, Geneva, Switzerland; International Centre for Eye Health; National Institute for Health Research Biomedical Research Centre for Ophthalmology at Moorfields Eye Hospital NHS Foundation Trust and UCL Institute of Ophthalmology, London, UK; International Centre for Eye Health; School of Optometry and Vision Science, University of Auckland, Auckland, New Zealand; Ministerio de Salud de la Nación, Buenos Aires, Argentina; Fundacion Oftalmologica Hugo Nano, Buenos Aires; University of New South Wales, Sydney, Australia; Gyal Yum Kesang Choden National Eye Center, JDWNRH, Thimphu, Bhutan; University of Burundi, Bujumbura, Burundi; The Affiliated Hospital of Yunnan University, Kunming, China; The Affiliated Eye Hospital of Nanchang University, Nanchang; Instituto Cubano de Oftalmologia Ramon Pando Ferrer, Habana, Cuba; Hospital Dr Elías Santana, Santo Domingo, Dominican Republic; Fundacion Oftalmologica del Valle, Yaruquí, Ecuador; Magrabi Foundation, Cairo, Egypt; Universidad de El Salvador, San Salvador, El Salvador; Jimma University, Jimma, Ethiopia; University of Bonn, Bonn, Germany; Augenzentrum Mittelmosel-Hunsrück, Traben-Trarbach; Visualiza, Guatemala City, Guatemala; Hospital San Felipe, Tegucigalpa, Honduras; The Chinese University of Hong Kong, Hong Kong; Semmelweis University, Budapest, Hungary; Sadguru Netra Chikitsalya, Chitrakoot, India; PBMA’s H V Desai Eye Hospital, Pune; Hasanuddin University Hospital, Makassar, Indonesia; Ministry of Education, Culture, Research, and Technology, Jakarta; Cicendo Eye Hospital, Bandung; Tehran University of Medical Sciences, Tehran, Iran; Noor Dubai Foundation, Dubai, United Arab Emirates, Jordan; University of Nairobi, Nairobi, Kenya; Kenyatta National Hospital, Nairobi; Ministry of Public Health, Antananarivo, Madagascar; Kamuzu College of Health Sciences, Blantyre, Malawi; Selayang Hospital, Selayang, Malaysia; Maldives Allied Health Council, Male, Maldives; Instituto de la Visión Hospital La Carlota, Montemorelos, Mexico; Help Me See, New York, USA; London School of Hygiene & Tropical Medicine, London, UK; State University of Medicine and Pharmacy Nicolae Testemitanu, Chişinău, Moldova; The Mongolian National University of Medical Sciences, Ulan Bator, Mongolia; Tilganga Institute of Ophthalmology, Kathmandu, Nepal; Seva Foundation, Kathmandu; Nepal Netra Jyoti Sangh, Kathmandu; International Agency for Prevention of Blindness - South East Asia, Kathmandu; Usmanu Danfodiyo University Teaching Hospital, Sokoto, Nigeria; Pakistan Institute of Community Ophthalmology, Peshawar, Pakistan; St John’s Eye Hospital, Jerusalem, Palestine; Pan American Health Organization, Panama City, Panama; Fundación Vision, Fernando de la Mora, Paraguay; Fundación Vision, Fernando de la Mora; Clinica Global Laser, Lima, Peru; Cataract Foundation Philippines, Bacolod City, Philippines; Rwanda International Institute of Ophthalmology, Kigali, Rwanda; Bin Rushd Eye Specialist Center, Riyadh, Saudi Arabia; Ministry of Health, Dodoma, Tanzania; Makerere University, Kampala, Uganda; Ministry of Health, Lusaka, Zambia

## Abstract

**Background:**

Cataract is the leading cause of blindness globally. Effective cataract surgical coverage (eCSC) measures the number of people in a population who have been operated on for cataract, and had a good outcome, as a proportion of all people operated on or requiring surgery. Therefore, eCSC describes service access (ie, cataract surgical coverage, [CSC]) adjusted for quality. The 74th World Health Assembly endorsed a global target for eCSC of a 30-percentage point increase by 2030. To enable monitoring of progress towards this target, we analysed Rapid Assessment of Avoidable Blindness (RAAB) survey data to establish baseline estimates of eCSC and CSC.

**Methods:**

In this secondary analysis, we used data from 148 RAAB surveys undertaken in 55 countries (2003–21) to calculate eCSC, CSC, and the relative quality gap (% difference between eCSC and CSC). Eligible studies were any version of the RAAB survey conducted since 2000 with individual participant survey data and census population data for people aged 50 years or older in the sampling area and permission from the study’s principal investigator for use of data. We compared median eCSC between WHO regions and World Bank income strata and calculated the pooled risk difference and risk ratio comparing eCSC in men and women.

**Findings:**

Country eCSC estimates ranged from 3·8% (95% CI 2·1–5·5) in Guinea Bissau, 2010, to 70·3% (95% CI 65·8–74·9) in Hungary, 2015, and the relative quality gap from 10·8% (CSC: 65·7%, eCSC: 58·6%) in Argentina, 2013, to 73·4% (CSC: 14·3%, eCSC: 3·8%) in Guinea Bissau, 2010. Median eCSC was highest among high-income countries (60·5% [IQR 55·6–65·4]; n=2 surveys; 2011–15) and lowest among low-income countries (14·8%; [IQR 8·3–20·7]; n=14 surveys; 2005–21). eCSC was higher in men than women (148 studies pooled risk difference 3·2% [95% CI 2·3–4·1] and pooled risk ratio of 1·20 [95% CI 1·15–1·25]).

**Interpretation:**

eCSC varies widely between countries, increases with greater income level, and is higher in men. In pursuit of 2030 targets, many countries, particularly in lower-resource settings, should emphasise quality improvement before increasing access to surgery. Equity must be embedded in efforts to improve access to surgery, with a focus on underserved groups.

**Funding:**

Indigo Trust, Peek Vision, and Wellcome Trust.

## Introduction

Effective service coverage indicators are WHO’s preferred measure for countries to monitor progress towards universal health coverage.^1^ Effective coverage indicators capture data on the coverage of services in the population (a measure of access), as well as quality of care.^2^

Cataract is the leading cause of blindness globally^3^ and, as such, a key focus of eye health services. Treatment can improve quality of life and reduce poverty^4^ and, although there is regional variation, the cost-effectiveness of cataract surgery compares favourably to that of other surgical procedures in low-income and middle-income countries (LMICs).^5,6^ Cataract surgical coverage (CSC) is a service coverage indicator that measures the number of people in a population who have been operated on for cataract as a proportion of all people operated on or still requiring surgery. CSC has been reported from eye health surveys for more than two decades.^7^ Effective CSC (eCSC)—first defined in 2017—uses postoperative visual acuity to quality-correct cataract surgical coverage.^8^ The use of a clinical measure of quality is a key strength of eCSC because effective coverage indicators in most other areas of health care rely on proxy quality measures.^1,9^

Given the large unmet need for cataract surgery—a cost-effective intervention with a standardised calculation method—eCSC represents an important indicator to monitor progress in eye care. In recognition of this need, member states at the 74th World Health Assembly endorsed a new global target for eCSC (a 30-percentage point increase) to be achieved by 2030.^10^ Further, the resolution called for countries with a baseline eCSC of 70% or higher to strive for universal coverage. Beyond eye health, eCSC will be considered for a revised Sustainable Development Goal monitoring framework in 2025.^11^

Here we present the most comprehensive analysis of eCSC to date. We have updated sex-disaggregated CSC and eCSC estimates to establish a baseline of cataract service coverage and quality, using data collected with the most commonly used population-based eye health survey methodology worldwide: the Rapid Assessment of Avoidable Blindness (RAAB).^12^ An update to the groups of people included in the calculation of CSC and eCSC is explained here for the first time.

## Methods

### Data source

In this secondary analysis, data to estimate CSC and eCSC were collected from population-based surveys conducted using the standardised RAAB survey methodology or its predecessor the Rapid Assessment of Cataract Surgical Services, hereafter both referred to as RAABs.^12,13^ The variables of interest for CSC and eCSC (visual acuity, lens status, and cause of vision impairment) have remained consistent across both versions. RAABs sample only the population aged 50 years and older (who have the vast majority of cataract-related vision impairment) and have an historical emphasis on district-level surveys in LMICs. The use of a certified RAAB trainer scheme allows for a high level of quality assurance and comparability between surveys. The RAAB repository records metadata for surveys undertaken since 2000, with datasets stored where available.

Ethical approval for analysis of RAAB repository data was obtained from the London School of Hygiene & Tropical Medicine Ethics Committee (25471).

### Study selection

We identified potentially eligible surveys from the RAAB repository. Eligible studies were any version of the RAAB survey conducted since 2000 with a complete dataset available (ie, individual participant survey data and census population data showing male and female 5-year age-sex group counts for people aged ≥50 years in the sampling area) and permission from the study’s principal investigator for use of data.

### Outcome variables

CSC estimates people with operated cataract (aphakia or pseudophakia) as a proportion of people with operated cataract plus people with cataract and best corrected visual acuity (BCVA) worse than a specified surgical threshold (sometimes referred to as operable cataract; panel). eCSC estimates people with operated cataract attaining a defined level of postoperative presenting visual acuity (ie, with optical correction, if available) as a proportion of the same denominator (panel). CSC and eCSC can be reported using different visual acuity thresholds for surgery (cataract surgical threshold) and postoperative good outcome. Due to having the most data, our main analysis used 6/18 for both thresholds unless stated otherwise.

### Data analysis

All data management and analyses were conducted using R software (version 4.2.1). We calculated CSC and eCSC for total populations and women and men separately from all available RAAB surveys. In cases where both eyes were operated on, presenting visual acuity in the better eye was used to define postoperative visual acuity. The gap between CSC and eCSC values can be considered a quality gap; we calculated the relative quality gap for each study as (CSC–eCSC)/CSC, with lower values reflecting better quality of cataract surgical services.

We post-stratified all estimates to the age structure of the population aged 50 years and older in the survey area using population data (eg, census data) applicable to the same study area and time period of the survey. Corresponding 5-year age-sex group population counts were provided by principal investigators at the same time as their survey data. We calculated age-adjusted, sex-adjusted estimates (age-adjusted only for sex-disaggregated estimates) from individual participant level data using the numerators and denominators for CSC and eCSC in age-sex strata (male and female for each of the 50–59, 60–69, 70–79, and ≥80 year age groups) in the survey data, multiplied those by an adjustment factor per stratum (number examined in the sample divided by the number in the population) and calculated adjusted coverage from the summed outputs. To account for RAAB’s two-stage cluster sampling strategy,^12^ 95% CIs were calculated from standard errors adjusted for the clustering of the sample and the variability between clusters of the denominator using formula six from Bennett and colleagues.^15^ This approach, which is consistent with RAAB’s standardised automated reporting analysis, gives more conservative standard errors than the exact binomial formula by including parameters to account for clustering and differences in cluster sizes.

Where two or more surveys were available from a country, only one estimate was used according to a predetermined decision tree based on sampling frame representativeness (national or subnational) and the time in years since the studies were completed (appendix p 1); this is referred to as the country estimate. National surveys were either a single survey (typically in smaller countries) or a series of subnational surveys designed for national coverage. Where there was no national estimate available, we pooled any two or more subnational surveys done within 3 years of the most recent single survey. More recent studies and studies with nationally representative sampling frames (either a single survey or a pooled series of subnational surveys) were prioritised. We combined subnational estimates from the same country using an inverse variance weighted average, which was calculated using the metagen command from the meta package in R.^16^

We used country estimates to compare the median, IQR, and range for surveys grouped geographically (by WHO region) and by income level (using World Bank country income level;^17^ high, upper middle, lower middle, and low). We used survey estimates in random effects meta-analysis of the risk difference and risk ratio comparing men and women. We also compared eCSC estimates over time where comparable sampling frames had been used in repeat studies.

### Role of the funding source

The funders had no role in the study design, data collection, data analysis, data interpretation, or in the writing of the report.

## Results

We identified 148 surveys for inclusion in this analysis (appendix pp 2–7), which comprised 24 national and 124 subnational samples undertaken in 55 countries between 2003 and 2021. All six WHO regions were represented. Many more studies were available from 2010 onwards (n=115) than for 2000–09 (n=33). The 148 datasets were 44% of the 335 RAABs known to have been done as of April 1, 2022; data for the remaining 187 surveys had not been shared with the RAAB repository or permission for use in secondary analysis had not been granted by the study principal investigator (appendix p 8).

**Figure 1:**
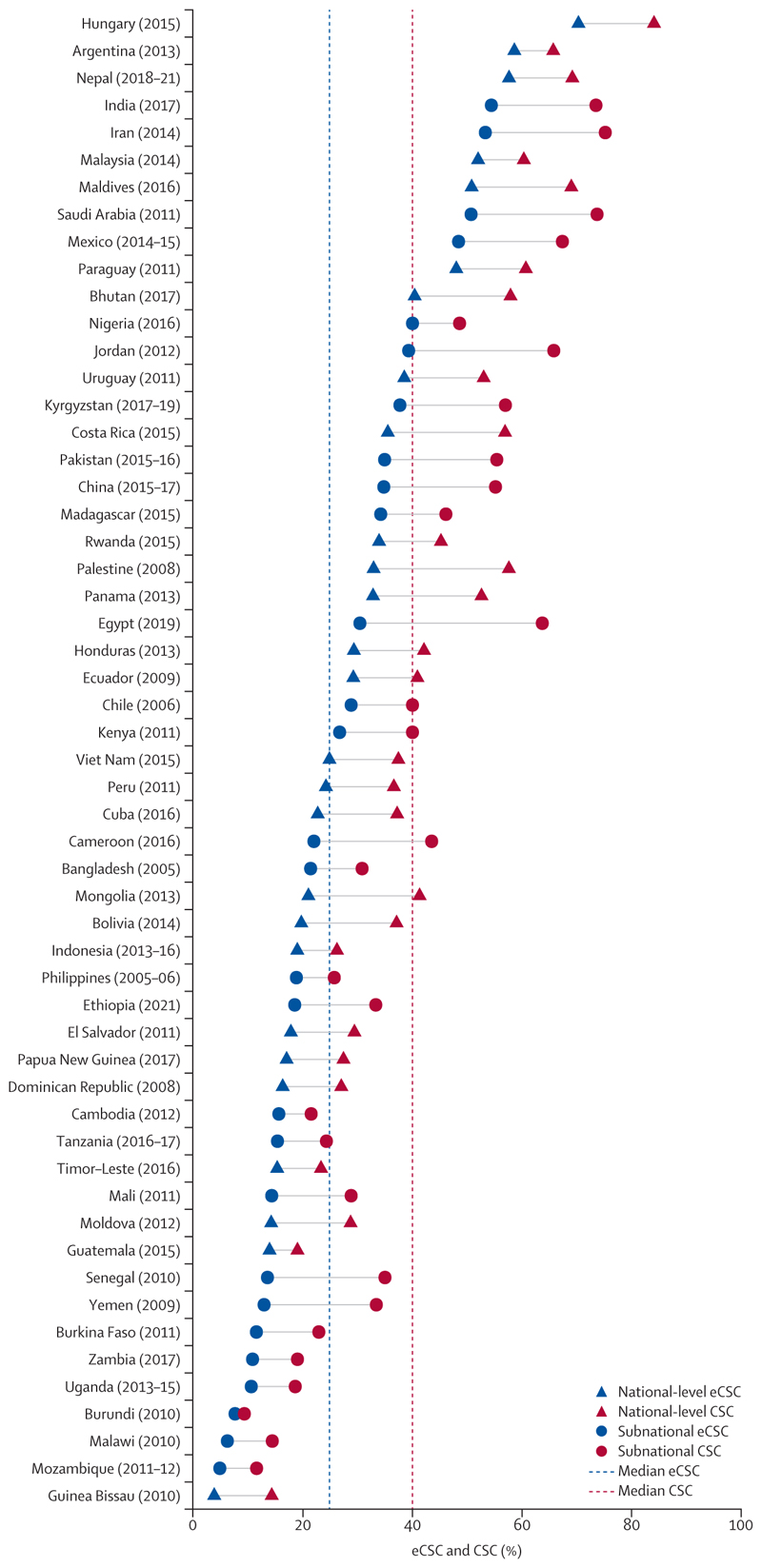
Country estimates of eCSC and CSC Cataract surgical threshold of less than 6/18 and 6/18 threshold for a good outcome. CSC=cataract surgical coverage. eCSC=effective cataract surgical coverage.

The number of participants in each survey ranged from 1171 to 6482 (total 468 852; median 2995·5 [IQR 2418·8–3765·5), and the number having had bilateral or unilateral cataract surgery (with <6/18 BCVA in the unoperated eye) in each survey ranged from five to 692 (total 22 150; median 113·0 [IQR 59·0–177·3]). 105 of 148 studies were used to generate 55 country estimates (figure 1; appendix pp 9–11). Individual subnational estimates were pooled for 14/54 country results (appendix p 12).

The highest eCSC estimate (6/18 threshold for operable cataract and good outcome) was from Hungary (2015) at 70·3% (95% CI 65·8–74·9) and the lowest was from Guinea Bissau (2010) at 3·8% (95% CI 2·1–5·5; figure 1). From all 55 included countries, the median eCSC estimate was 24·8% (IQR 15·5–38·1) and the median CSC estimate was 40·0% (IQR 27·2–57·3). There was variation in the quality gap between CSC and eCSC across countries, as indicated by the length of the line between point estimates in figure 1. The smallest relative quality gap was 10·8% in Argentina (2013; CSC 65·7%, eCSC 58·6%), and the largest relative gap was in Guinea Bissau (2010) at 73·4% (CSC 14·3%, eCSC 3·8%; appendix pp 9–11). 15 countries had CSC higher than 50% but a relative quality gap of more than 25% (appendix p 13).

**Figure 2:**
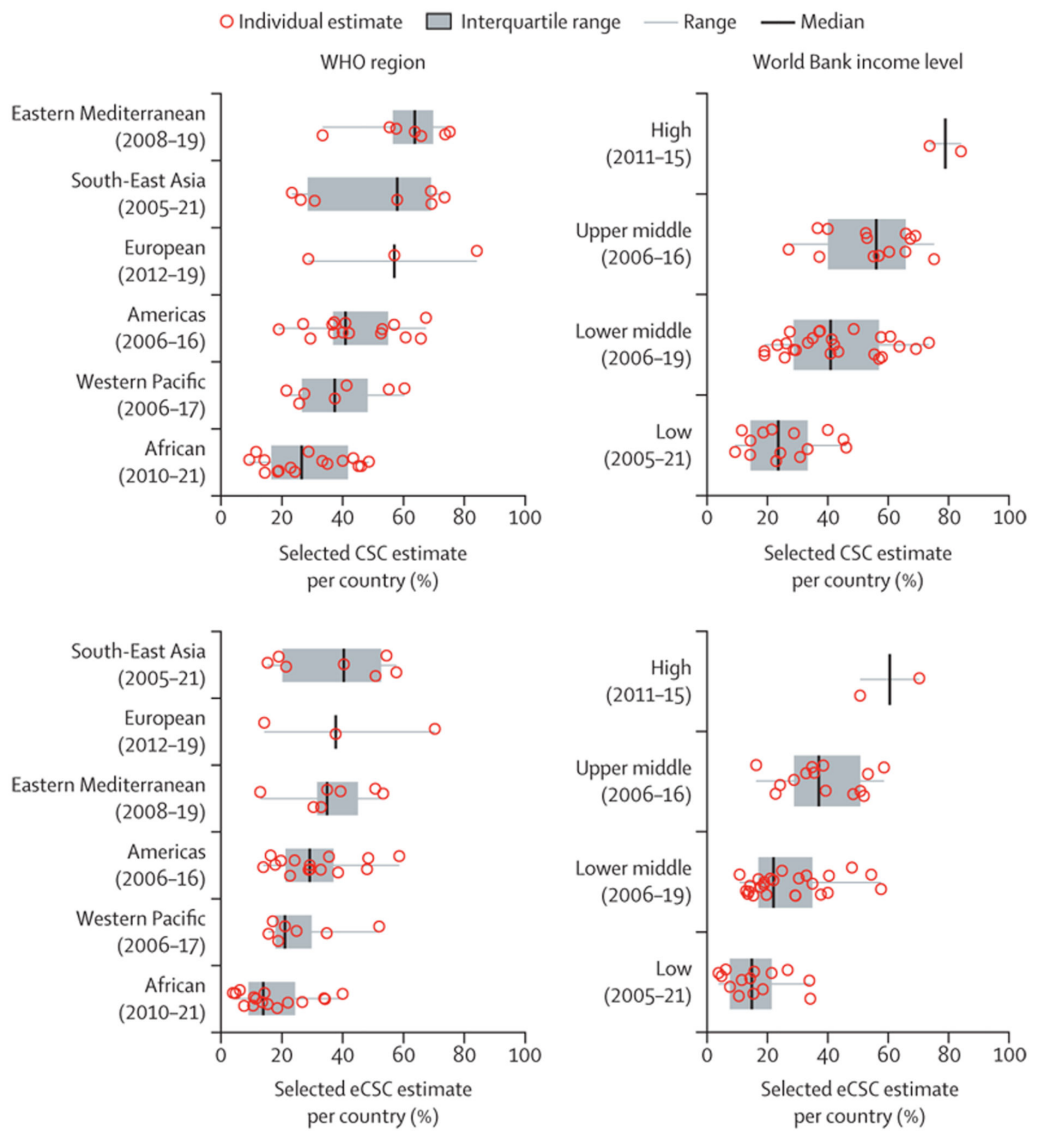
Country estimates of eCSC and CSC by WHO region and World Bank income strata Cataract surgical threshold of less than 6/18 and 6/18 threshold for a good outcome. CSC=cataract surgical coverage. eCSC=effective cataract surgical coverage.

By World Bank income strata, median eCSC was highest in the high-income group (60·5% [IQR 55·6–65·4]; n=2 surveys; 2011–15) and got progressively lower moving down to 14·8% (IQR 8·3–20·7; n=14 surveys; 2005–21) in the low-income group (figure 2). The median regional eCSC was highest in the South-East Asia region (40·4% [IQR 20·2–52·6]; n=7 surveys; 2005–21) and in the European region (37·7% [IQR 26·0–54·0]; n=3 surveys; 2012–19) and lowest in the African region (13·9% [IQR 9·8–23·2]; n=16 surveys; 2010–21; figure 2). The Eastern Mediterranean region had the highest median CSC at 63·7% (IQR 56·5–69·8; n=7 surveys; 2008–19), but the quality gap meant the median eCSC in the region was lower than in the South-East Asia region, or the European region (figure 2).

There were four settings for which data were available from two timepoints. In Bhutan, eCSC improved from 25·3% (95% CI 19·3–31·3) in the first national survey in 2009 to 40·4% (95% CI 35·2–45·5) in the second in 2017. Pooled eCSC estimates from two series of subnational surveys done in Nepal in 2008–10 (n=10 surveys) and 2018–21 (n=7 surveys) showed that eCSC increased from 38·1% (95% CI 30·3–45·9) to 57·6% (95% CI 50·4–64·8) across the decade. The relative quality gap in Bhutan remained similar (30·3% to 30·2%), and in Nepal it decreased from 25·5% to 16·7%. At a subnational level, in Nuevo León state, Mexico, eCSC increased from 30·7% (95% CI 25·0–36·4%) to 54·1% (95% CI 49·1–59·1%) between 2005 and 2014 (the relative quality gap decreased from 33·4% to 25·3%), and in Koulikoro region, Mali, eCSC did not increase significantly between surveys in 2008 (10·8% [95% CI 8·0–13·6%]) and 2011 (14·3% [95% CI 9·4–19·2%]).

**Figure 3:**
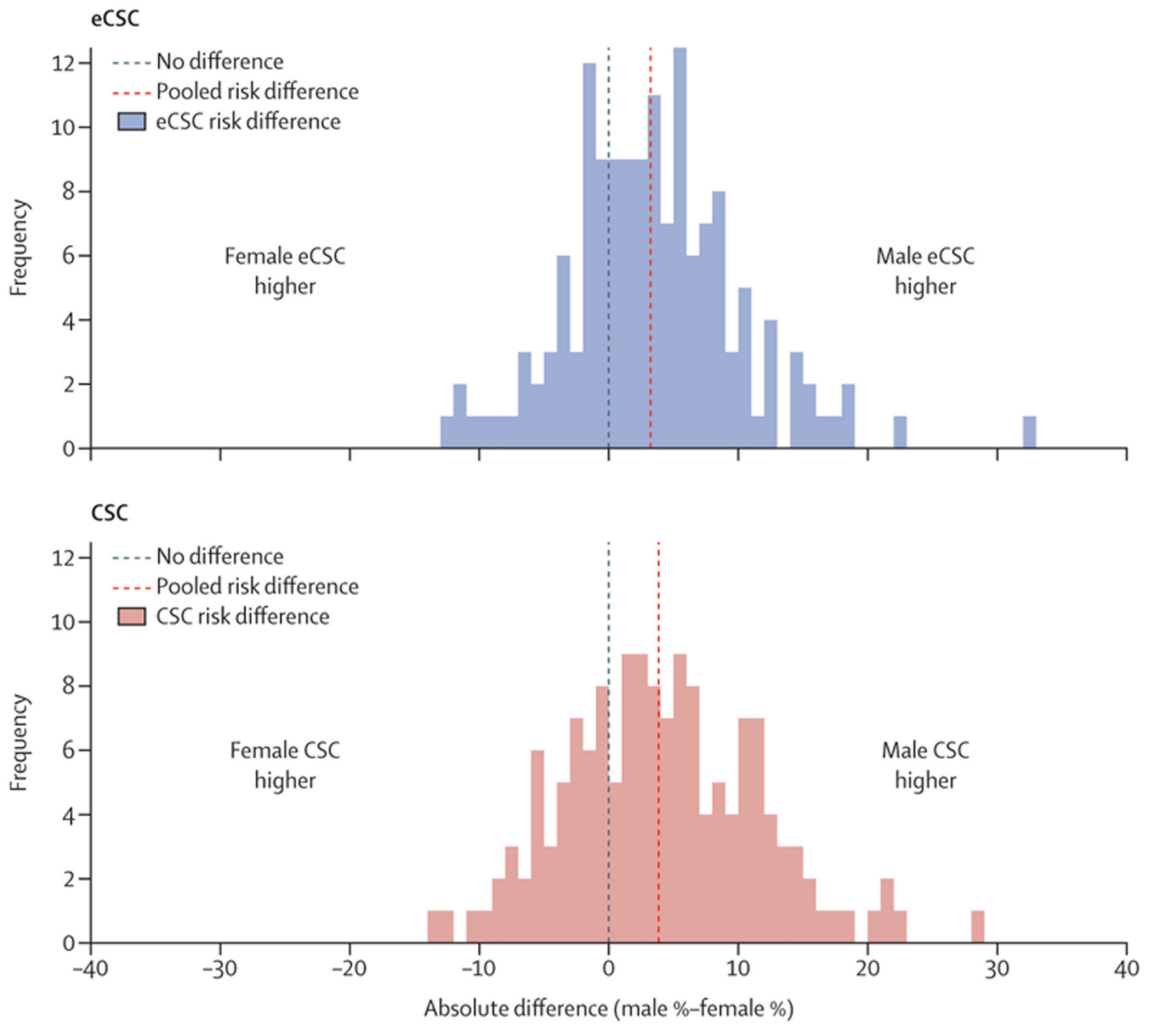
The absolute difference in male and female eCSC and CSC Cataract surgical threshold of less than 6/18 and 6/18 threshold for a good outcome. Risk differences calculated for 148 surveys. CSC=cataract surgical coverage. eCSC=effective cataract surgical coverage.

eCSC was higher in men than women (148 studies pooled risk difference 3·2% [95% CI 2·3–4·1]; figure 3; and pooled risk ratio of 1·20 [95% CI 1·15–1·25]). For CSC, the absolute difference (3·9% [95% CI 2·7–5·0]; figure 3) and prevalence ratio (1·15 [95% CI 1·11–1·19]) in favour of men were similar to effective coverage (appendix pp 14–21). There was no evidence of an absolute or relative pooled difference in male and female eCSC or CSC in the region of the Americas (20 studies, 2003–16) or the Europe region (four studies, 2012–19) with the CIs including null (no difference; appendix pp 14–21).

**Figure 4:**
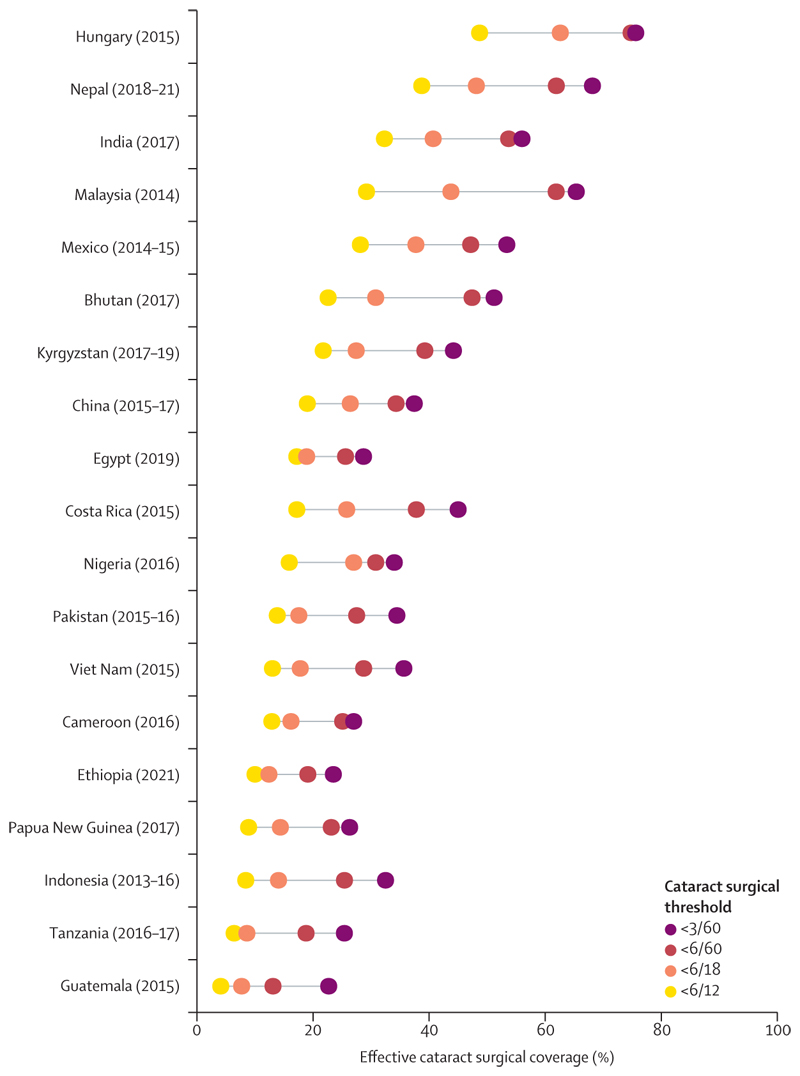
Range of effective cataract surgical coverage across four cataract surgical thresholds 6/12 threshold for a good outcome.

Data to calculate eCSC at the 6/12 visual acuity threshold were available for 63 studies from 19 countries. For these country estimates (determined using the same decision-tree process outlined above), there was considerable variation in eCSC (using 6/12 as a good outcome) across four cataract surgical thresholds (figure 4). This variation was largest in Malaysia (2014) where eCSC was 65·4% using a surgical threshold of less than 3/60, 61·9% at a threshold of less than 6/60, 43·8% at a threshold of less than 6/18 and 29·2% at a threshold of less than 6/12. At the worse than 6/12 cataract surgical threshold, no country had eCSC greater than 50% and four had eCSC under 10%.

## Discussion

We conducted a secondary analysis of 148 population-based eye health surveys (from 55 countries, between 2003 and 2021) to generate new estimates of eCSC. This analysis serves as a basis to monitor progress towards the global eCSC target, a 30-percentage point increase by 2030, that was endorsed at the 74th World Health Assembly, while also highlighting important data gaps to address through future surveys.^10^ We used an updated, more inclusive definition of CSC and eCSC (panel). The median eCSC across 55 countries was 24·8% and median CSC was 40·0%. We found wide variation in eCSC by country and a gradient of increasing eCSC with increasing World Bank country income level stratum, reflecting the tendency for greater resources and subsequent cataract service output in countries with higher income.^6,18^ The South-East Asia and European regions had the highest eCSC, and the African region had the lowest. The Eastern Mediterranean region had the highest crude coverage but had a lower effective coverage than South-East Asia and European regions.

These results reveal that there can be a large gap between CSC and eCSC and highlight the need for countries to consider both components of eCSC—ie, access and quality—as they set about meeting their 2030 target. The range of interventions needed to increase quality and access or quantity of cataract services can be quite different. The relative quality gap and the level of crude coverage could help determine the relative emphasis providers might place on quality improvement and scaling up services. For example, countries with a relative quality gap of less than perhaps 25% might want to focus on improving access while maintaining or improving quality. In contrast, countries with a larger quality gap of 25% or higher might choose to particularly invest in quality improvement initiatives before focusing on actions to increase access or output. Our results highlighted 15 countries with CSC of 50% or higher that could make considerable progress towards the 2030 eCSC target by improving quality alone.

Countries can gain additional context by considering recent facility-based surgical outcome data and their cataract surgical rate (number of operations per million people per year). Contemporary outcome monitoring is important because eCSC reflects the results of all surgeries received by participants, some of which might have been decades earlier using superseded techniques, or after which postoperative comorbidities unrelated to surgery had developed. Cataract surgical rate is commonly low in LMICs;^6,18^ surgeries might be more frequently done on eyes with more advanced cataract and comorbidities, meaning good postoperative visual outcomes are less probable. Moreover, if individual surgeons are performing relatively few cataract operations, they might not gain enough experience to improve and maintain skills. Here, surgeons should not be disincentivised to operate but should be supported to deliver quality outcomes while increasing output. For example, trial evidence from multiple African countries indicates that a training intervention package combining surgical simulation with deliberate sustained practice can substantially improve patient safety and surgeon productivity.^19^

Although quality is captured in eCSC through postoperative visual acuity (ie, effectiveness), there are other important components of quality health care^20^ not captured (eg, safety, efficiency, people centredness, and timeliness) that should be considered in quality improvement initiatives, particularly in high-resource settings where visual outcomes are already routinely good.^21^ Measuring effectiveness using presenting visual acuity rather than best-corrected acuity reflects patients’ lived experience of postoperative visual outcomes. Residual refractive error can be caused by limitations in availability of ocular biometry and a range of intraocular lens powers, particularly in low-resource settings. Alongside investment in surgical equipment, training, and point-of-care monitoring of surgical outcomes, increasing the availability and affordability of refractive and optical services, including through better integration with surgical services, will help reduce residual refractive error.^21^ This last strategy offers the opportunity to improve effective refractive error coverage^14,22^ for distance and near vision as well as eCSC.^6^

There have been encouraging examples of improvements in eCSC, which countries can draw on, with increases ranging from 15 to 23 percentage points over 8 to 10 years. For example, over the period Nepal increased its eCSC by 20 percentage points, the proportion of surgeries with an intraocular lens increased from 86% to 98%. A further driver of improvement was a major funder of outreach cataract surgery in the country making biometry mandatory in 2007, meaning people were much more likely to receive the most appropriate intraocular lens and therefore achieve better postoperative visual acuity (unpublished data).

To overcome inequities in cataract vision impairment that are ubiquitous within countries, efforts to improve access to good-quality cataract services should focus on historically underserved groups that might include vulnerable populations (eg, low socioeconomic position or low social support) and people living in rural communities.^6^ For example, in Australia, a country with high-quality services, a national survey estimated that the eCSC for non-Indigenous Australians (89%) was substantially higher than that for Indigenous Australians (52%).^23^ Due to data availability, our analysis focused on sex disparities. Our findings were consistent with previous analyses which found that men were more likely to have accessed cataract surgery than women in various LMICs globally, in south Asia, and in India (all measured by CSC),^24–26^ while a study from Latin America found no overall difference in eCSC by sex in the areas surveyed.^27^

A recent exercise to identify the most relevant eye health indicators for universal health coverage recommended that indicators be disaggregated by sex, place of residence, socioeconomic position, and disability status.^28^ Once the population groups with lowest eCSC are identified, countries can implement strategies to improve access for these groups. Unfortunately, evidence on how to do this is currently limited, but includes patient counselling, transport provision, and free surgery.^29–31^ Financial protection, the third dimension of universal health coverage,^32^ is an important strategy to improve access to health services. Although cataract surgery is a cost-effective intervention, cost is frequently reported as a barrier to access,^33^ and the availability of financial protection for cataract surgery is largely unknown.^6^ WHO has developed a package of eye care interventions to support countries in planning and budgeting for integrating eye care interventions into their health systems.^34^ This package recommends that high-priority eye care interventions, including cataract surgery, should be included in service packages covered by pre-paid pooled financing.

WHO anticipates that the shift to a 6/12 threshold for a good outcome and cataract surgical threshold will stimulate quality improvement and encourage providers to offer surgery earlier with less vision loss. At the same time, WHO proposed that countries also report effective coverage at the cataract surgical thresholds most applicable to their context.^14^ We found a marked reduction in eCSC (at the 6/12 threshold for a good outcome) with lower cataract surgical thresholds. Our results highlight that there are still settings where people with cataract blindness have been unable to access services and, here, the relevance of monitoring eCSC at multiple cataract surgical thresholds is clear. Countries might (arguably rightly) prioritise using limited resources to provide services to people with more vision loss, meaning less progress will be observed at the 6/18 and 6/12 thresholds.^35^

In some settings, service providers might find patients with early cataract easier to reach and treat effectively, thereby increasing effective coverage. However, this approach will only serve to widen inequalities between population subgroups. Preoperative visual acuity among cataract surgery patients is a recommended indicator in WHO’s eye care indicator menu and could be monitored to assess if surgical output is tailored to populations at greater risk of cumulative disadvantage.^36,37^

The strengths of this analysis of historical survey data include the breadth of RAAB survey data available compared to previous studies.^8,27^ All studies were conducted using a comparable RAAB sampling and examination protocol, were overseen by centrally accredited survey trainers, and supported by standardised software, providing assurance of the internal and external validity of the estimates. We used individual participant level data to estimate new cluster-adjusted, age-adjusted, and sex-adjusted eCSC and CSC in place of the crude estimates previously reported and presented an estimate per country according to a decision tree that prioritised newer, nationally representative data.

Our study also had several limitations. First, estimates were unavailable for most countries. Second, data were only available from two high-income countries. Because high-income countries would be expected to have higher eCSC, our regional results probably underestimate the true eCSC. For example, a recent survey in Australia^23^ found an eCSC of 89% at the 6/12 threshold among the non-Indigenous population, which suggests the true gap between high-income and low-income countries might be wider than we report here. Third, it is possible that in low-income regions, countries with population-based data might have more mature eye care services than countries that have no population-based data. Therefore, our median value for such regions might overestimate effective coverage if the situation in countries with no data is in fact less favourable. Fourth, there were 187 surveys from 25 additional countries listed on the RAAB repository that were not available for inclusion in this analysis. These studies were similar to included studies in terms of being national or subnational and decade of completion, and as such we believe those included are a reasonable representation of all RAABs done to date. Fifth, we included RAABs done between 2003 and 2021 and, as such, estimates were not directly comparable across countries or regions. Finally, just under half of the country estimates (27/55) were nationally representative; the remainder report results from subnational areas which might underestimate or overestimate effective coverage at the national level depending on whether services in the subnational area are stronger or weaker than the national average.

A 30-percentage point global increase in eCSC by 2030 is an ambitious target, which, if adequately supported by investment in services, could stimulate necessary improvements in cataract surgical quality and coverage, particularly in LMICs. In most such countries, quality improvement should be prioritised before increasing population access to surgery, but strategies to improve access and affordability for traditionally underserved groups are also required. More population-based surveys are needed to monitor eCSC through to 2030 with an emphasis on greater geographical coverage of data gaps, particularly in the European region, the Eastern Mediterranean region, and the region of the Americas.

## Supplementary Material

Supplementary Material

## Data Availability

140 of the datasets used in this study are available online to registered users of the RAAB repository website (https://www.raab.world/) and eight datasets are available by request from survey principal investigators via the corresponding author. Code used to calculate eCSC and CSC from RAAB data is available at https://github.com/raabteam/raab7-analysis.

## Research in context

### Evidence before this study

We searched PubMed in January, 2021, without language or date restrictions, for reviews and secondary analyses of effective cataract surgical coverage or cataract surgical coverage using the terms: “effective cataract surg* coverage” and “cataract surg* coverage”. Effective cataract surgical coverage (eCSC) was first described and reported in 2017 alongside the relative quality gap between eCSC and cataract surgical coverage (CSC). This analysis used crude estimates from 20 Rapid Assessment of Avoidable Blindness (RAAB) surveys of the population aged 50 years and older from 20 countries done between 2005 and 2013. For the 20 surveys, median CSC was 53·7% and median eCSC was 36·7% (using an operable cataract visual acuity threshold of <6/60 and a good outcome threshold of 6/18); men had better eCSC than women (median risk difference 4·6% [IQR 0·5–7·1], odds ratio 1·3 [95% CI 1·0–1·5]). In addition, primary studies are increasingly reporting eCSC. For example, a comprehensive national eye health survey conducted in Australia in 2016 found eCSC (cataract vision impairment threshold of <6/12 and a good outcome threshold of 6/12) was 89% among the non-Indigenous population and 52% among Indigenous Australians. Four systematic reviews with meta-analysis have found women less likely to access cataract surgical services (measured by CSC) in surveys from low-income and middle-income countries globally, in south Asia, and in India. Another systematic review of studies conducted in Latin America found no difference in CSC by sex in the areas surveyed.

### Added value of this study

Compared with the first report of eCSC in 2017, our study considerably increases the breadth of data used to calculate eCSC, including many RAAB survey datasets with no previous eCSC output. We used 148 RAAB surveys (done from 2003 to 2021) and provided estimates for 55 countries. We used population data contemporary to the surveys to report age-adjusted and sex-adjusted estimates in place of crude estimates used previously. We demonstrated differences in eCSC by geography (WHO region) and country income level (World Bank income strata). We confirmed findings of sex inequality in eCSC and identified regional variation in sex inequality in eCSC, previously shown in analyses of CSC only. Additionally, this Article sets out a modification to the CSC and eCSC calculations recently endorsed at a WHO Technical Meeting. The indicators now include all people with unilateral met and unmet need for cataract surgery to alleviate vision impairment.

### Implications of all the available evidence

eCSC has been low in many settings between 2003 and 2021 and the World Health Assembly target for 2030 is ambitious but not impossible. We found examples from three countries where eCSC had increased by 15 to 23 percentage points over 8–10 years. In many settings, particularly low-income and middle-income countries, considerable investment in cataract services will be required to see improvement in line with the global target. This study emphasises the relative quality gap between CSC and eCSC as a metric for countries to consider when planning cataract service interventions and whether quality improvement should be prioritised or might be done in parallel to improving access to services. We identified that gender inequity in eCSC needs to be addressed in most settings and recognise that, in addition to women, other underserved groups should be prioritised to see equitable increases in eCSC. Finally, improved access to historical data and new population-based surveys are required to monitor progress.

## *Panel*: Visual acuity thresholds in Rapid Assessment of Avoidable Blindness (RAAB) and an updated effective cataract surgical coverage (eCSC) definition

RAAB survey protocols up to RAAB5 screened visual acuity to the 6/18 level (moderate vision impairment), and subsequent versions (2013 onward) also screened visual acuity at the 6/12 level (mild vision impairment). Cataract surgical thresholds have historically been reported as: worse than 3/60 (blindness), worse than 6/60 (severe vision impairment), and worse than 6/18. In 2021, after consultation with member states and eye care stakeholders, WHO proposed that eCSC should also be reported against a cataract surgical threshold of worse than 6/12 (mild vision impairment). The recommendation also included updating the definition of a good surgical outcome from 6/18 or better to 6/12 or better.^14^ Here, we outline an update to the previously defined calculations,^8 14^ endorsed at a WHO Technical Meeting in July, 2022.

Although cataract is often a bilateral condition, people with cataract in one eye and vision impairment from another cause in the other eye can still benefit from surgery. The recent update now includes two groups of people that were historically excluded from cataract surgical coverage (CSC) and eCSC calculations:

- People with operated cataract in one eye (people included in the x and a variables) are included in the numerator with any cause of vision impairment in the other eye (determined using best corrected visual acuity [BCVA])
- People with an unmet need for surgery (people included in the z variable) are included in the denominator if they have bilateral cataract or unilateral cataract with any cause of vision impairment in the other eye (determined using BCVA)

This update gives a more complete picture of met and unmet need for cataract services, while also being more people-centred by not excluding anyone with vision impairment from cataract.

**Calculation for CSC**

$$\text{CSC}=\frac{\left(x+y\right)}{\left(x+y+z\right)}$$

where *x* is individuals with unilateral operated cataract (regardless of visual acuity in the operated eye) and vision impairment (using BCVA*) in the other eye, *y* is individuals with bilateral operated cataract (regardless of visual acuity in the operated eyes), and *z* is individuals with vision impairment (using BCVA*) in both eyes with cataract as the main cause of vision impairment in one or both eyes.†

**Calculation for eCSC**

$$\text{eCSC}=\frac{\left(a+b\right)}{\left(x+y+z\right)}$$

Where *a* is individuals with unilateral operated cataract attaining a specified threshold of postoperative presenting visual acuity in the operated eye, who have vision impairment (using BCVA*) in the other eye and *b* is individuals with bilateral operated cataract attaining a specified threshold of postoperative presenting visual acuity in at least one eye.†

*According to the cataract surgical threshold used in the estimate (<6/12, <6/18, <6/60, or <3/60). †Cases of couched eyes are excluded from the count of x, y, a, and b.

## References

[1] WHO and International Bank for Reconstruction and Development, The World Bank (2017). Tracking universal health coverage: 2017 Global Monitoring Report.

[2] Ng M, Fullman N, Dieleman JL, Flaxman AD, Murray CJ, Lim SS (2014). Effective coverage: a metric for monitoring universal health coverage. PLoS Med.

[3] Steinmetz JD, Bourne RRA, Briant PS (2021). Causes of blindness and vision impairment in 2020 and trends over 30 years, and prevalence of avoidable blindness in relation to VISION 2020: the Right to Sight: an analysis for the Global Burden of Disease Study. Lancet Glob Health.

[4] Danquah L, Kuper H, Eusebio C (2014). The long term impact of cataract surgery on quality of life, activities and poverty: results from a six year longitudinal study in Bangladesh and the Philippines. PLoS One.

[5] Chao TE, Sharma K, Mandigo M (2014). Cost-effectiveness of surgery and its policy implications for global health: a systematic review and analysis. Lancet Glob Health.

[6] Burton MJ, Ramke J, Marques AP (2021). The Lancet Global Health Commission on Global Eye Health: vision beyond 2020. Lancet Glob Health.

[7] Limburg H, Foster A (1998). Cataract surgical coverage: an indicator to measure the impact of cataract intervention programmes. Community Eye Health.

[8] Ramke J, Gilbert CE, Lee AC, Ackland P, Limburg H, Foster A (2017). Effective cataract surgical coverage: an indicator for measuring quality-of-care in the context of universal health coverage. PLoS One.

[9] Boerma T, AbouZahr C, Evans D, Evans T (2014). Monitoring intervention coverage in the context of universal health coverage. PLoS Med.

[10] WHO (2020). Integrated people-centred eye care, including preventable vision impairment and blindness: report by the Director General.

[11] UN Vision for Everyone: accelerating action to achieve the Sustainable Development Goals.

[12] Kuper H, Polack S, Limburg H (2006). Rapid assessment of avoidable blindness. Community Eye Health.

[13] Limburg H, Kumar R, Indrayan A, Sundaram KR (1997). Rapid assessment of prevalence of cataract blindness at district level. Int J Epidemiol.

[14] Keel S, Müller A, Block S (2021). Keeping an eye on eye care: monitoring progress towards effective coverage. Lancet Glob Health.

[15] Bennett S, Woods T, Liyanage WM, Smith DL (1991). A simplified general method for cluster-sample surveys of health in developing countries. World Health Stat Q.

[16] Olivoto T, Lúcio A (2020). metan: an R package for multi-environment trial analysis. Methods Ecol Evol.

[17] The World Bank (2021). The world by income and region.

[18] Wang W, Yan W, Fotis K (2016). Cataract surgical rate and socioeconomics: a global study. Invest Ophthalmol Vis Sci.

[19] Dean WH, Gichuhi S, Buchan JC (2021). Intense simulation-based surgical education for manual small-incision cataract surgery: the ophthalmic learning and improvement initiative in cataract surgery randomized clinical trial in Kenya, Tanzania, Uganda, and Zimbabwe. JAMA Ophthalmol.

[20] WHO (2018). Delivering quality health services: a global imperative.

[21] Yoshizaki M, Ramke J, Zhang JH (2021). How can we improve the quality of cataract services for all? A global scoping review. Clin Exp Ophthalmol.

[22] McCormick I, Mactaggart I, Bastawrous A, Burton MJ, Ramke J (2020). Effective refractive error coverage: an eye health indicator to measure progress towards universal health coverage. Ophthalmic Physiol Opt.

[23] Keel S, Xie J, Foreman J, Taylor HR, Dirani M (2018). Population-based assessment of visual acuity outcomes following cataract surgery in Australia: the National Eye Health Survey. Br J Ophthalmol.

[24] Lewallen S, Mousa A, Bassett K, Courtright P (2009). Cataract surgical coverage remains lower in women. Br J Ophthalmol.

[25] Ye Q, Chen Y, Yan W (2020). Female gender remains a significant barrier to access cataract surgery in south Asia: a systematic review and meta-analysis. J Ophthalmol.

[26] Prasad M, Malhotra S, Kalaivani M, Vashist P, Gupta SK (2020). Gender differences in blindness, cataract blindness and cataract surgical coverage in India: a systematic review and meta-analysis. Br J Ophthalmol.

[27] Reis T, Lansingh V, Ramke J, Silva JC, Resnikoff S, Furtado JM (2021). Cataract as a cause of blindness and vision impairment in Latin America: progress made and challenges beyond 2020. Am J Ophthalmol.

[28] McCormick I, Mactaggart I, Resnikoff S (2021). Eye health indicators for universal health coverage: results of a global expert prioritisation process. Br J Ophthalmol.

[29] Ramke J, Petkovic J, Welch V (2017). Interventions to improve access to cataract surgical services and their impact on equity in low- and middle-income countries. Cochrane Database Syst Rev.

[30] Ramke J, Evans JR, Gilbert CE (2018). Reducing inequity of cataract blindness and vision impairment is a global priority, but where is the evidence?. Br J Ophthalmol.

[31] Mailu EW, Virendrakumar B, Bechange S, Jolley E, Schmidt E (2020). Factors associated with the uptake of cataract surgery and interventions to improve uptake in low- and middle-income countries: a systematic review. PLoS One.

[32] WHO (2016). Strategizing national health in the 21st century: a handbook.

[33] Aboobaker S, Courtright P (2016). Barriers to cataract surgery in Africa: a systematic review. Middle East Afr J Ophthalmol.

[34] WHO (2022). Package of eye care interventions.

[35] Jolley E, Cumaio M, Vilanculos A (2022). Changes in eye health and service coverage in Nampula, Mozambique between 2011 and 2018. Ophthalmic Epidemiol.

[36] Sabherwal S, Kuyyadiyil S, Tomar VPS (2021). A multicentric cross-sectional study measuring the equity of cataract surgical services in three high-volume eyecare organizations in North India: equitable cataract surgical rate as a new indicator. Indian J Ophthalmol.

[37] WHO (2022). Eye care indicator menu (ECIM): a tool for monitoring strategies and actions for eye care provision.

