# Supplementary material for: Effective cataract surgical coverage in adults aged 50 years and older: estimates from population-based surveys in 55 countries
Source: Lancet Glob Health. Author manuscript; Available in PMC 2025 Oct 23. (PMC7618287; doi:10.1016/S2214-109X(22)00419-3)
Supplement: Supplementary Material [file EMS209641-supplement-Supplementary_Material.pdf]

THE LANCET
Global Health

Supplementary appendix

This appendix formed part of the original submission and has been peer reviewed. We post it as supplied by the authors.

Supplement to: McCormick I, Butcher R, Evans JR. Effective cataract surgical coverage in adults aged 50 years and older: estimates from population-based surveys in 55 countries. *Lancet Glob Health* 2022; published online October 11. https://doi.org/10.1016/S2214-109X(22)00419-3.

Supplementary material

Effective cataract surgical coverage in adults aged 50 years and older: estimates from population-based surveys in 55 countries

Contents

1. Decision tree for inclusion of survey data presented per country *1*

2. List of Rapid Assessment of Avoidable Blindness data sources *2*

3. A comparison of RAAB surveys since 2000 available and unavailable for inclusion in estimates of CSC and eCSC *8*

4. Country eCSC, CSC and relative quality gap estimates at the 6/18 threshold for a good outcome and operable cataract *9*

5. Pooled eCSC values used for country estimates at the 6/18 threshold for a good outcome and operable cataract *12*

6. Country estimates of effective cataract surgical coverage and cataract surgical coverage (6/18 threshold for good outcome and operable cataract) grouped by relative quality gap categories *13*

7. Forest plots of absolute (A) and relative (B) difference in male and female eCSC at the 6/18 threshold for a good outcome and operable cataract (by WHO region and overall) *14*

8. Forest plots of absolute (A) and relative (B) difference in male and female CSC at the 6/18 threshold for a good outcome and operable cataract (by WHO region and overall) *18*

1. Decision tree for inclusion of survey data presented per country

To summarise the available data per country with the most relevant estimates, we presented a single sex-disaggregated estimate of CSC and eCSC. Where only a single RAAB was available, we used its findings. Some estimates were calculated from a nationally representative sample, others derived from a subnational survey. Where two or more estimates were available per country, we followed the steps outlined below to arrive at the selected values presented.

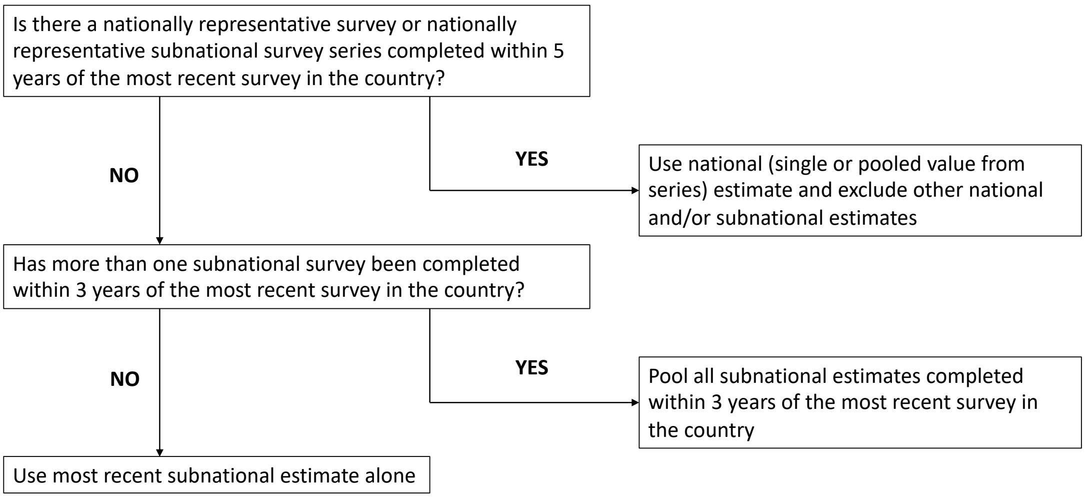

2. List of Rapid Assessment of Avoidable Blindness data sources

WHO Region	Country	Year	Survey title	Level	Sample size	Response rate (%)	Proportion female (%)	Data source
Africa	Burkina Faso	2011	Burkina Faso, West Central (2011)	Subnational	2299	94·1	60·9	www.raab.world
Africa	Burundi	2010	Burundi, Ngozi and Kayanza (2010)	Subnational	3879	95·0	58·5	www.raab.world
Africa	Cameroon	2016	Cameroon, Centre, Yaounde (2016)	Subnational	4080	91·9	58·7	www.raab.world
Africa	Ethiopia	2021	Ethiopia, Oromia, Jimma (2021)	Subnational	3850	100·0	44·5	www.raab.world
Africa	Guinea Bissau	2010	Guinea Bissau (2010)	National	2900	99·0	55·4	www.raab.world
Africa	Kenya	2005	Kenya, Rift Valley, Nakuru (2005)	Subnational	3750	92·7	52·6	www.raab.world
Africa	Kenya	2007	Kenya, Eastern, Embu (2007)	Subnational	3403	99·2	55·1	www.raab.world
Africa	Kenya	2007	Kenya, Rift Valley, Kericho (2007)	Subnational	2546	95·0	50·1	www.raab.world
Africa	Kenya	2011	Kenya, Coast, Kwale (2011)	Subnational	3250	96·1	50·7	www.raab.world
Africa	Madagascar	2015	Madagascar, Analamanga (2015)	Subnational	3800	94·9	59·6	www.raab.world
Africa	Malawi	2010	Malawi, South-western health zone (2010)	Subnational	3583	95·7	62·2	www.raab.world
Africa	Mali	2008	Mali, Koulikoro (2008)	Subnational	2600	93·8	56·4	www.raab.world
Africa	Mali	2011	Mali, Koulikoro (2011)	Subnational	2300	96·8	57·9	www.raab.world
Africa	Mozambique	2011	Mozambique, Nampula (2011)	Subnational	3050	96·9	54·4	www.raab.world
Africa	Mozambique	2012	Mozambique, Sofala (2012)	Subnational	3599	94·1	58·7	www.raab.world
Africa	Nigeria	2016	Nigeria, Sokoto, Wurno (2016)	Subnational	2700	89·1	45·8	www.raab.world
Africa	Rwanda	2006	Rwanda, South-west (2006)	Subnational	2250	98·0	53·5	www.raab.world
Africa	Rwanda	2015	Rwanda (2015)	National	5194	97·5	61·0	www.raab.world
Africa	Senegal	2010	Senegal, Fatick (2010)	Subnational	2600	96·7	57·9	www.raab.world
Africa	Senegal	2010	Senegal, Kaolack (2010)	Subnational	2900	97·7	57·6	www.raab.world
Africa	Tanzania	2016	Tanzania, Morogoro (2016)	Subnational	3000	96·1	55·4	www.raab.world
Africa	Tanzania	2017	Tanzania, Singida (2017)	Subnational	3850	96·7	56·2	www.raab.world
Africa	Uganda	2011	Uganda, Western, Ntungamo (2011)	Subnational	3840	96·4	57·8	www.raab.world
Africa	Uganda	2012	Uganda, Central, Mubende (2012)	Subnational	3850	96·9	52·6	www.raab.world
Africa	Uganda	2013	Uganda, Western, Hoima (2013)	Subnational	3862	99·1	50·8	www.raab.world

Africa	Uganda	2015	Uganda, Northern, Karamoja (2015)	Subnational	3850	96·8	61·8	www.raab.world
Africa	Zambia	2017	Zambia, Muchinga (2017)	Subnational	3600	97·3	54·9	www.raab.world
Americas	Argentina	2003	Argentina, Buenos Aires (urban) (2003)	Subnational	4600	93·5	55·9	www.raab.world
Americas	Argentina	2013	Argentina (2013)	National	4098	92·0	55·1	www.raab.world
Americas	Bolivia	2014	Bolivia (2014)	National	3344	95·5	57·3	www.raab.world
Americas	Chile	2006	Chile, Bio Bio (2006)	Subnational	3000	97·2	58·2	www.raab.world
Americas	Costa Rica	2015	Costa Rica (2015)	National	3255	76·6	63·0	Contact PI
Americas	Cuba	2016	Cuba (2016)	National	3919	99·3	58·8	www.raab.world
Americas	Dominican Republic	2008	Dominican Republic (2008)	National	3995	96·9	51·5	www.raab.world
Americas	Ecuador	2009	Ecuador (2009)	National	4200	95·5	54·1	www.raab.world
Americas	El Salvador	2011	El Salvador (2011)	National	3800	89·4	59·5	www.raab.world
Americas	Guatemala	2004	Guatemala, Southwest (2004)	Subnational	4900	98·1	62·4	www.raab.world
Americas	Guatemala	2015	Guatemala (2015)	National	3850	97·7	59·4	www.raab.world
Americas	Honduras	2013	Honduras (2013)	National	3150	95·2	59·4	www.raab.world
Americas	Mexico	2005	Mexico, Nuevo Leon (2006)	Subnational	3780	99·6	56·9	www.raab.world
Americas	Mexico	2010	Mexico, Chiapas, Central, Highland and Frailesca (2010)	Subnational	3249	86·8	59·3	www.raab.world
Americas	Mexico	2014	Mexico, Nuevo Leon (2014)	Subnational	5219	92·5	61·2	www.raab.world
Americas	Mexico	2015	Mexico, Queretaro (2015)	Subnational	6300	94·2	53·3	www.raab.world
Americas	Panama	2013	Panama (2013)	National	4200	98·2	54·5	www.raab.world
Americas	Paraguay	2011	Paraguay (2011)	National	3000	95·4	53·1	www.raab.world
Americas	Peru	2011	Peru (2011)	National	5000	97·0	58·5	www.raab.world
Americas	Uruguay	2011	Uruguay (2011)	National	3956	94·3	57·9	www.raab.world
Eastern Mediterranean	Egypt	2019	Egypt, Sohag (2019)	Subnational	4078	98·9	40·8	www.raab.world
Eastern Mediterranean	Iran	2009	Iran, Tehran, Varamin (2009)	Subnational	3000	94·0	54·9	www.raab.world
Eastern Mediterranean	Iran	2014	Iran, Kurdistan (2014)	Subnational	3465	92·4	51·7	www.raab.world
Eastern Mediterranean	Jordan	2012	Jordan, Irbid (2012)	Subnational	3778	96·2	49·5	www.raab.world
Eastern Mediterranean	Pakistan	2015	Pakistan, FATA, Peshawar (2015)	Subnational	3185	96·8	49·0	www.raab.world

Eastern Mediterranean	Pakistan	2016	Pakistan, Khyber Pakhtunkhwa, Mansehra (2016)	Subnational	3588	97·8	45·0	www.raab.world
Eastern Mediterranean	Pakistan	2016	Pakistan, Khyber Pakhtunkhwa, Swabi (2016)	Subnational	3526	99·7	44·6	www.raab.world
Eastern Mediterranean	Palestine	2008	Palestine (2008)	National	3800	94·2	54·8	www.raab.world
Eastern Mediterranean	Saudi Arabia	2011	Saudi Arabia, Makkah, Al Taif (2011)	Subnational	3300	93·0	46·1	www.raab.world
Eastern Mediterranean	Yemen	2009	Yemen, Amran (2009)	Subnational	1948	91·8	46·5	www.raab.world
Europe	Hungary	2015	Hungary (2015)	National	3675	95·9	63·9	www.raab.world
Europe	Kyrgyzstan	2017	Kyrgyzstan, South-west (2017)	Subnational	3000	96·6	58·5	www.raab.world
Europe	Kyrgyzstan	2019	Kyrgyzstan, North-east (2019)	Subnational	3000	95·2	60·4	www.raab.world
Europe	Moldova	2012	Moldova (2012)	National	2693	99·8	67·5	www.raab.world
South-East Asia	Bangladesh	2005	Bangladesh, Khulna, Satkhira (2005)	Subnational	5295	91·9	55·6	www.raab.world
South-East Asia	Bhutan	2009	Bhutan (2009)	National	4100	98·7	51·9	www.raab.world
South-East Asia	Bhutan	2017	Bhutan (2017)	National	5050	98·4	51·8	www.raab.world
South-East Asia	India	2008	India, Uttar Pradesh, Chitrakoot (2008)	Subnational	1320	93·0	53·8	www.raab.world
South-East Asia	India	2017	India, Maharashtra, Pune (urban) (2017)	Subnational	3600	89·5	55·3	www.raab.world
South-East Asia	Indonesia	2013	Indonesia, South Sulawesi (2013)	Subnational	4381	99·2	62·4	www.raab.world
South-East Asia	Indonesia	2014	Indonesia, West Java (2014)	Subnational	3000	94·7	62·5	www.raab.world
South-East Asia	Indonesia	2015	Indonesia, Bali (2015)	Subnational	3050	99·1	56·7	www.raab.world
South-East Asia	Indonesia	2015	Indonesia, Central Java (2015)	Subnational	3000	99·4	58·6	www.raab.world
South-East Asia	Indonesia	2015	Indonesia, East Java (2015)	Subnational	2842	99·3	61·7	www.raab.world
South-East Asia	Indonesia	2015	Indonesia, Jakarta (2015)	Subnational	3050	98·3	68·8	www.raab.world
South-East Asia	Indonesia	2016	Indonesia, Maluku (2016)	Subnational	2800	97·9	59·5	www.raab.world
South-East Asia	Indonesia	2016	Indonesia, North Sulawesi (2016)	Subnational	2850	97·7	61·9	www.raab.world
South-East Asia	Indonesia	2016	Indonesia, North Sumatra (2016)	Subnational	2950	98·9	58·7	www.raab.world
South-East Asia	Indonesia	2016	Indonesia, Nusa Tenggara Timur (2016)	Subnational	2846	97·1	53·9	www.raab.world
South-East Asia	Indonesia	2016	Indonesia, South Kalimantan (2016)	Subnational	3000	96·4	59·5	www.raab.world
South-East Asia	Indonesia	2016	Indonesia, South Sumatra (2016)	Subnational	3049	99·0	63·7	www.raab.world
South-East Asia	Indonesia	2016	Indonesia, West Papua (2016)	Subnational	2886	95·1	52·4	www.raab.world
South-East Asia	Indonesia	2016	Indonesia, West Sumatra (2016)	Subnational	3050	97·5	62·2	www.raab.world

South-East Asia	Maldives	2016	Maldives (2016)	National	3100	97·4	59·2	www.raab.world
South-East Asia	Nepal	2008	Nepal, Bagmati (2008)	Subnational	2050	93·1	54·6	www.raab.world
South-East Asia	Nepal	2008	Nepal, Janakpur (2008)	Subnational	1800	94·7	54·0	www.raab.world
South-East Asia	Nepal	2008	Nepal, Karnali (2008)	Subnational	1197	97·8	44·8	www.raab.world
South-East Asia	Nepal	2008	Nepal, Seti and Mahakali (2008)	Subnational	2751	91·3	53·5	www.raab.world
South-East Asia	Nepal	2009	Nepal, Bheri (2009)	Subnational	3050	98·1	51·3	www.raab.world
South-East Asia	Nepal	2009	Nepal, Koshi (2009)	Subnational	3050	94·9	53·6	www.raab.world
South-East Asia	Nepal	2009	Nepal, Mechi (2009)	Subnational	3050	99·7	48·3	www.raab.world
South-East Asia	Nepal	2009	Nepal, Sagarmatha (2009)	Subnational	3050	95·5	52·9	www.raab.world
South-East Asia	Nepal	2010	Nepal, Dhaulagiri (2010)	Subnational	3000	99·7	53·6	www.raab.world
South-East Asia	Nepal	2010	Nepal, Rapti (2010)	Subnational	2988	97·4	52·6	www.raab.world
South-East Asia	Nepal	2015	Nepal, Narayani (2015)	Subnational	5000	95·4	52·4	www.raab.world
South-East Asia	Nepal	2018	Nepal, Lumbini Province (2018)	Subnational	5642	98·9	55·2	Contact PI
South-East Asia	Nepal	2019	Nepal, Karnali Province (2019)	Subnational	4075	97·9	53·0	Contact PI
South-East Asia	Nepal	2019	Nepal, Bagmati Province (2019)	Subnational	5739	95·3	57·9	Contact PI
South-East Asia	Nepal	2020	Nepal, Province 1 (2020)	Subnational	4231	97·4	53·9	Contact PI
South-East Asia	Nepal	2020	Nepal, Province 2 (2020)	Subnational	4075	99·5	53·2	Contact PI
South-East Asia	Nepal	2020	Nepal, Gandaki Province (2020)	Subnational	4794	98·4	54·8	Contact PI
South-East Asia	Nepal	2021	Nepal, Sudurpashchim Province (2021)	Subnational	4607	99·1	56·4	Contact PI
South-East Asia	Timor-Leste	2016	Timor-Leste (2016)	National	3350	97·1	51·5	www.raab.world
Western Pacific	Cambodia	2007	Cambodia (2007)	National	6000	98·4	62·8	www.raab.world
Western Pacific	Cambodia	2012	Cambodia, Takeo (2012)	Subnational	4650	96·2	65·6	www.raab.world
Western Pacific	China	2006	China, Yunnan, Kunming (2006)	Subnational	2760	93·8	59·7	www.raab.world
Western Pacific	China	2007	China, Jiangxi, Gao'an (2007)	Subnational	5000	94·0	55·1	www.raab.world
Western Pacific	China	2007	China, Jiangxi, Wanzai (2007)	Subnational	3000	95·4	52·7	www.raab.world
Western Pacific	China	2007	China, Jiangxi, Xingan (2007)	Subnational	4000	95·9	55·6	www.raab.world
Western Pacific	China	2008	China, Yunnan, Luliang (2008)	Subnational	3000	94·7	52·7	www.raab.world
Western Pacific	China	2010	China, Hainan (rural) (2010)	Subnational	6800	95·3	53·5	www.raab.world

Western Pacific	China	2010	China, Inner Mongolia, Shangdu (2010)	Subnational	2000	98·8	50·4	www.raab.world
Western Pacific	China	2010	China, Inner Mongolia, Tuoketuo (2010)	Subnational	2098	96·4	52·5	www.raab.world
Western Pacific	China	2011	China, Sichuan, Dechang (2011)	Subnational	1749	98·5	53·3	www.raab.world
Western Pacific	China	2011	China, Sichuan, Mianning (2011)	Subnational	2850	98·8	65·2	www.raab.world
Western Pacific	China	2012	China, Guangdong, Chaonan (2012)	Subnational	3700	94·2	59·9	www.raab.world
Western Pacific	China	2012	China, Yunnan, Jianchuan (2012)	Subnational	2100	95·6	57·4	www.raab.world
Western Pacific	China	2012	China, Yunnan, Lancang (2012)	Subnational	2550	94·8	53·6	www.raab.world
Western Pacific	China	2015	China, Xinjiang, Altay and Tacheng (2015)	Subnational	4150	95·8	54·5	www.raab.world
Western Pacific	China	2017	China, Sichuan, Garze (2017)	Subnational	5000	95·3	56·2	www.raab.world
Western Pacific	Malaysia	2014	Malaysia, Johor and Melaka (2014)	Subnational	2500	94·8	55·3	www.raab.world
Western Pacific	Malaysia	2014	Malaysia, Kelantan, Terengganu and Pahang (2014)	Subnational	2500	98·0	58·6	www.raab.world
Western Pacific	Malaysia	2014	Malaysia, Kuala Lumpur, Putrajaya, Selangor and Negeri Sembilan (2014)	Subnational	2500	91·3	55·9	www.raab.world
Western Pacific	Malaysia	2014	Malaysia, Perlis, Kedah, Penang and Perak (2014)	Subnational	2500	96·7	57·4	www.raab.world
Western Pacific	Malaysia	2014	Malaysia, Sabah and Labuan (2014)	Subnational	2500	95·4	55·3	www.raab.world
Western Pacific	Malaysia	2014	Malaysia, Sarawak (2014)	Subnational	2500	95·4	54·0	www.raab.world
Western Pacific	Mongolia	2013	Mongolia (2013)	National	4040	99·7	59·9	www.raab.world
Western Pacific	Papua New Guinea	2017	Papua New Guinea, Coastal (2017)	Subnational	1250	99·4	49·5	www.raab.world
Western Pacific	Papua New Guinea	2017	Papua New Guinea, Highlands (2017)	Subnational	1250	96·4	40·5	www.raab.world
Western Pacific	Papua New Guinea	2017	Papua New Guinea, Islands (2017)	Subnational	1250	94·3	49·2	www.raab.world
Western Pacific	Papua New Guinea	2017	Papua New Guinea, National Capital District (2017)	Subnational	1250	95·4	55·1	www.raab.world
Western Pacific	Philippines	2005	Philippines, Western Visayas, Negros (2005)	Subnational	3649	76·0	58·4	www.raab.world
Western Pacific	Philippines	2006	Philippines, Western Visayas, Antique (2006)	Subnational	3842	82·7	61·7	www.raab.world
Western Pacific	Vietnam	2010	Vietnam, Son La (2010)	Subnational	3146	97·6	57·1	www.raab.world
Western Pacific	Vietnam	2011	Vietnam, Thanh Hoa (2011)	Subnational	5000	97·5	59·4	www.raab.world
Western Pacific	Vietnam	2012	Vietnam, Nghe An (2012)	Subnational	5000	92·3	57·5	www.raab.world
Western Pacific	Vietnam	2012	Vietnam, Quang Nam (2012)	Subnational	4950	98·8	61·3	www.raab.world
Western Pacific	Vietnam	2015	Vietnam, Bac Ninh (2015)	Subnational	1974	95·7	59·1	www.raab.world
Western Pacific	Vietnam	2015	Vietnam, Binh Duong (2015)	Subnational	1999	98·6	64·7	www.raab.world

Western Pacific	Vietnam	2015	Vietnam, Ca Mau (2015)	Subnational	1999	93·1	59·0	www.raab.world
Western Pacific	Vietnam	2015	Vietnam, Dien Bien (2015)	Subnational	1999	98·3	57·3	www.raab.world
Western Pacific	Vietnam	2015	Vietnam, Gia Lai (2015)	Subnational	2000	98·1	59·1	www.raab.world
Western Pacific	Vietnam	2015	Vietnam, Ha Tinh (2015)	Subnational	1983	99·4	63·5	www.raab.world
Western Pacific	Vietnam	2015	Vietnam, Lam Dong (2015)	Subnational	2000	98·3	61·4	www.raab.world
Western Pacific	Vietnam	2015	Vietnam, Nam Dinh (2015)	Subnational	1999	96·6	58·4	www.raab.world
Western Pacific	Vietnam	2015	Vietnam, Phu Tho (2015)	Subnational	1997	97·9	62·3	www.raab.world
Western Pacific	Vietnam	2015	Vietnam, Quang Ngai (2015)	Subnational	2000	99·8	64·6	www.raab.world
Western Pacific	Vietnam	2015	Vietnam, Quang Tri (2015)	Subnational	1998	100·0	60·5	www.raab.world
Western Pacific	Vietnam	2015	Vietnam, Tien Giang (2015)	Subnational	1999	91·8	61·5	www.raab.world
Western Pacific	Vietnam	2015	Vietnam, Tuyen Quang (2015)	Subnational	1999	96·0	58·9	www.raab.world
Western Pacific	Vietnam	2015	Vietnam, Vung Tau (2015)	Subnational	2000	96·7	55·5	www.raab.world

3. A comparison of RAAB surveys since 2000 available and unavailable for inclusion in estimates of CSC and eCSC

	Available		Unavailable		Total	
**WHO Region**	**N**	**%**	**N**	**%**	**N**	**%**
Africa	27	18·2	41	21·9	68	20·3
Americas	20	13·5	5	2·7	25	7·5
Eastern Mediterranean	10	6·8	21	11·2	31	9·3
Europe	4	2·7	2	1·1	6	1·8
South-East Asia	39	26·4	80	42·8	119	35·5
Western Pacific	48	32·4	38	20·3	86	25·7
**Decade of survey**						
2000-2009	33	22·3	64	34·2	97	29·0
2010-2019	110	74·3	114	61·0	224	66·9
2020-	5	3·4	9	4·8	14	4·2
**Survey representativeness**						
National	24	16·2	20	10·7	44	13·1
Subnational	124	83·8	167	89·3	291	86·9
**Total**	148	100·0	187	100·0	335	100·0

4. Country eCSC, CSC and relative quality gap estimates at the 6/18 threshold for a good outcome and operable cataract

Country	Level	Year	Participants examined (n)	Total cataract surgeries (n)	CSC <6/18 numerator (n)	Adjusted CSC <6/18 (%) [Estimate, lower 95% CI, upper 95% CI]			Adjusted eCSC <6/18 (%) [Estimate, lower 95% CI, upper 95% CI]			Relative quality gap (%)
**Africa**												
Burkina Faso	Subnational	2011	2163	97	69	22·9	17·6	28·1	11·5	7·8	15·2	49·8
Burundi	Subnational	2010	3655	10	5	9·3	0·0	21·8	7·6	0·0	19·7	18·3
Cameroon	Subnational	2016	3751	190	98	43·5	36·7	50·2	22·0	15·7	28·2	49·4
Ethiopia	Subnational	2021	3850	251	146	33·3	28·3	38·3	18·5	14·3	22·7	44·4
Guinea Bissau	National	2010	2870	104	65	14·3	9·4	19·2	3·8	2·1	5·5	73·4
Kenya	Subnational	2011	3124	299	159	40·0	34·5	45·5	26·7	22·7	30·7	33·2
Madagascar	Subnational	2015	3605	106	54	46·1	36·7	55·5	34·2	25·8	42·6	25·8
Malawi	Subnational	2010	3430	78	40	14·4	10·2	18·7	6·2	3·4	8·9	56·9
Mali	Subnational	2011	2226	173	119	28·8	21·5	36·1	14·3	9·4	19·2	50·3
Mozambique*	Subnational	2011-2012	6340	124	73	11·5	0·0	23·4	4·8	0·6	9·0	58·2
Nigeria	Subnational	2016	2405	117	83	48·6	41·1	56·1	40·0	33·3	46·7	17·7
Rwanda	National	2015	5065	141	67	45·2	36·5	53·9	33·9	25·6	42·3	25·0
Senegal*	Subnational	2010	5348	343	220	35·0	20·9	49·1	13·5	8·6	18·4	61·3
Tanzania*	Subnational	2016-2017	6606	299	184	24·3	17·3	31·2	15·3	11·9	18·8	36·8
Uganda*	Subnational	2013-2015	7554	199	116	18·6	15·4	21·8	10·6	7·3	13·8	43·2
Zambia	Subnational	2017	3502	92	58	19·0	14·6	23·3	10·8	7·5	14·1	43·2
**Median**						**26·5**			**13·9**			**43·8**
IQR						17·5-40·9			9·8-23·2			31·4-52·0
**Americas**												
Argentina	National	2013	3770	455	218	65·7	54·7	76·6	58·6	48·2	69·0	10·8
Bolivia	National	2014	3194	169	84	37·1	29·1	45·1	19·7	14·1	25·4	46·9
Chile	Subnational	2006	2915	140	77	40·0	33·3	46·6	28·8	22·4	35·1	28·0
Costa Rica	National	2015	2493	331	168	56·9	50·4	63·5	35·5	29·6	41·5	37·6
Cuba	National	2016	3890	314	184	37·2	32·8	41·6	22·7	18·7	26·6	39·0
Dominican Republic	National	2008	3873	172	92	27·0	21·5	32·4	16·3	11·8	20·8	39·6
Ecuador	National	2009	4012	444	235	40·9	36·3	45·5	29·2	24·9	33·5	28·6

El Salvador	National	2011	3399	216	113	29·4	24·2	34·7	17·8	13·2	22·3	39·5
Guatemala	National	2015	3760	100	51	19·0	13·9	24·0	13·9	9·9	17·8	26·8
Honduras	National	2013	2999	240	129	42·1	35·4	48·7	29·3	22·4	36·1	30·4
Mexico*	Subnational	2014-2015	10936	961	471	67·4	56·9	77·8	48·4	36·8	60·1	28·1
Panama	National	2013	4125	633	336	52·6	47·1	58·2	32·8	27·7	38·0	37·6
Paraguay	National	2011	2862	183	87	60·7	51·9	69·4	48	38·9	57·2	20·9
Peru	National	2011	4849	238	130	36·6	31·6	41·6	24·2	19·8	28·5	33·9
Uruguay	National	2011	3729	351	160	53·0	45·1	60·9	38·5	30·5	46·5	27·4
**Median**						**40·9**			**29·2**			**30·4**
IQR						36·9-55·0			21·2-37·0			27·7-38·3
**Eastern Mediterranean**												
Egypt	Subnational	2019	4033	1172	692	63·7	60·1	67·3	30·4	26·7	34·2	52·3
Iran	Subnational	2014	3203	746	372	75·2	70·5	79·9	53·3	47·9	58·6	29·1
Jordan	Subnational	2012	3636	531	277	65·8	61·0	70·6	39·3	33·7	45·0	40·3
Pakistan*	Subnational	2015-2016	10109	1328	761	55·4	36·0	74·8	34·9	11·8	58·0	37·0
Palestine	National	2008	3579	543	310	57·6	52·7	62·6	32·9	28·0	37·7	42·9
Saudi Arabia	Subnational	2011	3052	585	311	73·7	69·5	77·9	50·7	46·1	55·2	31·2
Yemen	Subnational	2009	1789	190	128	33·4	27·3	39·5	12·9	9·2	16·6	61·4
**Median**						**63·7**			**34·9**			**40·3**
IQR						56·5-69·8			31·7-45·0			34·1-47·6
**Europe**												
Hungary	National	2015	3523	738	346	84·1	80·2	88·0	70·3	65·8	74·9	16·4
Kyrgyzstan*	Subnational	2017-2019	5752	389	210	57·0	51·9	62·0	37·7	30·6	44·8	33·8
Moldova	National	2012	2687	102	55	28·7	21·8	35·5	14·2	8·9	19·6	50·5
**Median**						**57·0**			**37·7**			**33·8**
**IQR**						42·8-70·5			26·0-54·0			25·1-42·2
**South-East Asia**												
Bangladesh	Subnational	2005	4868	215	143	30·8	25·6	35·9	21·4	16·7	26·1	30·5
Bhutan	National	2017	4970	537	249	57·9	52·7	63·1	40·4	35·2	45·5	30·2
India	Subnational	2017	3221	1190	613	73·5	69·7	77·4	54·4	49·7	59·1	26·0
Indonesia*[$]	National	2013-2016	41850	1846	1021	26·2	20·1	32·3	19·0	14·1	23·8	27·6
Maldives	National	2016	3020	850	427	69·0	65·2	72·8	50·8	46·2	55·4	26·4
Nepal*	National	2018-2021	32500	6887	3473	69·2	62·3	76·1	57·6	50·4	64·8	16·7

Timor-Leste	National	2016	3253	137	82	23·3	17·5	29·2	15·3	10·7	20·0	34·3
**Median**						**57·9**			**40·4**			**27·6**
**IQR**						28·5-69·1			20·2-52·6			26·2-30·4
**Western Pacific**												
Cambodia	Subnational	2012	4471	372	209	21·5	18·3	24·8	15·6	12·7	18·5	27·4
China*	Subnational	2015-2017	8741	497	255	55·2	43·9	66·4	34·8	30·4	39·1	37·0
Malaysia*	National	2014	14289	1928	935	60·3	46·1	74·6	52·0	38·2	65·8	13·8
Mongolia	National	2013	4029	199	112	41·3	34·0	48·6	21·0	15·6	26·3	49·2
Papua New Guinea*	National	2017	4818	225	127	27·4	10·5	44·3	17·0	3·5	30·6	37·9
Philippines*	Subnational	2005-2006	5951	233	113	25·7	21·7	29·8	18·8	15·2	22·4	26·9
Vietnam*†	National	2015	27151	2816	1558	37·4	33·3	41·6	24·8	21·4	28·3	33·6
**Median**						**37·4**			**21·0**			**33·6**
**IQR**						26·6-48·2			17·9-29·8			27·2-37·4

*Pooled estimate calculated from two or more RAAB surveys based on the decision tree in appendix 1; $^{\$}$14 survey districts not nationally representative but purposively selected considering population size, geographic representation, available human resources and security; †14 survey districts not nationally representative but purposively selected to cover all regions

5. Pooled eCSC values used for country estimates at the 6/18 threshold for a good outcome and operable cataract

We pooled subnational estimates to produce a subset of the country estimates according to the decision tree in appendix 1. The table and figure below show pooled eCSC values and the subnational survey estimates for 14 countries.

Country	Level	Years	Pooled eCSC (95% confidence interval)	Contributing studies (N)	I^2 (%)	Cochran's Q test
China	Subnational	2015-2017	34·8 (30·4-39·1)	2	0·0	p=0·796
Indonesia	National	2013-2016	19·0 (14·1-23·8)	14	94·2	p<0·001
Kyrgyzstan	Subnational	2017-2019	37·7 (30·6-44·8)	2	51·9	p=0·149
Malaysia	National	2014	52·0 (38·2-65·8)	6	97·3	p<0·001
Mexico	Subnational	2014-2015	48·4 (36·8-60·1)	2	88·1	p=0·004
Mozambique	Subnational	2011-2012	4·8 (0·6-9·0)	2	84·3	p=0·012
Nepal	National	2018-2021	57·6 (50·4-64·8)	7	96·9	p<0·001
Pakistan	Subnational	2015-2016	34·9 (11·8-58·0)	3	98·6	p<0·001
Papua New Guinea	National	2017	17·0 (3·5-30·6)	4	90·2	p<0·001
Philippines	Subnational	2005-2006	18·8 (15·2-22·4)	2	0·0	p=0·388
Senegal	Subnational	2010	13·5 (8·6-18·4)	2	72·2	p=0·058
Tanzania	Subnational	2016-2017	15.3 (11·9-18·8)	2	41·2	p=0·192
Uganda	Subnational	2013-2015	10·6 (7·3-13·8)	2	31·0	p=0·229
Vietnam	National	2015	24·8 (21·4-28·3)	14	85·1	p<0·001

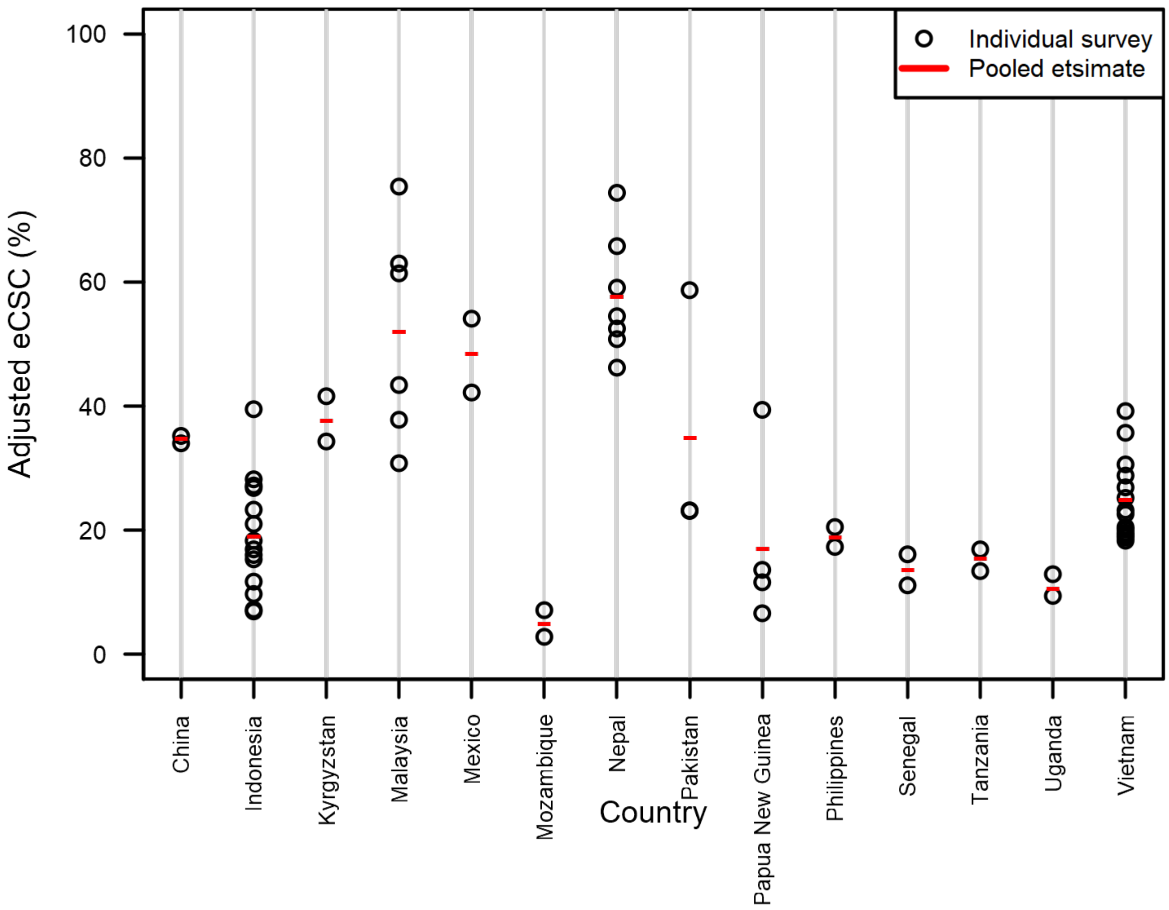

6. Country estimates of effective cataract surgical coverage and cataract surgical coverage (6/18 threshold for good outcome and operable cataract) grouped by relative quality gap categories

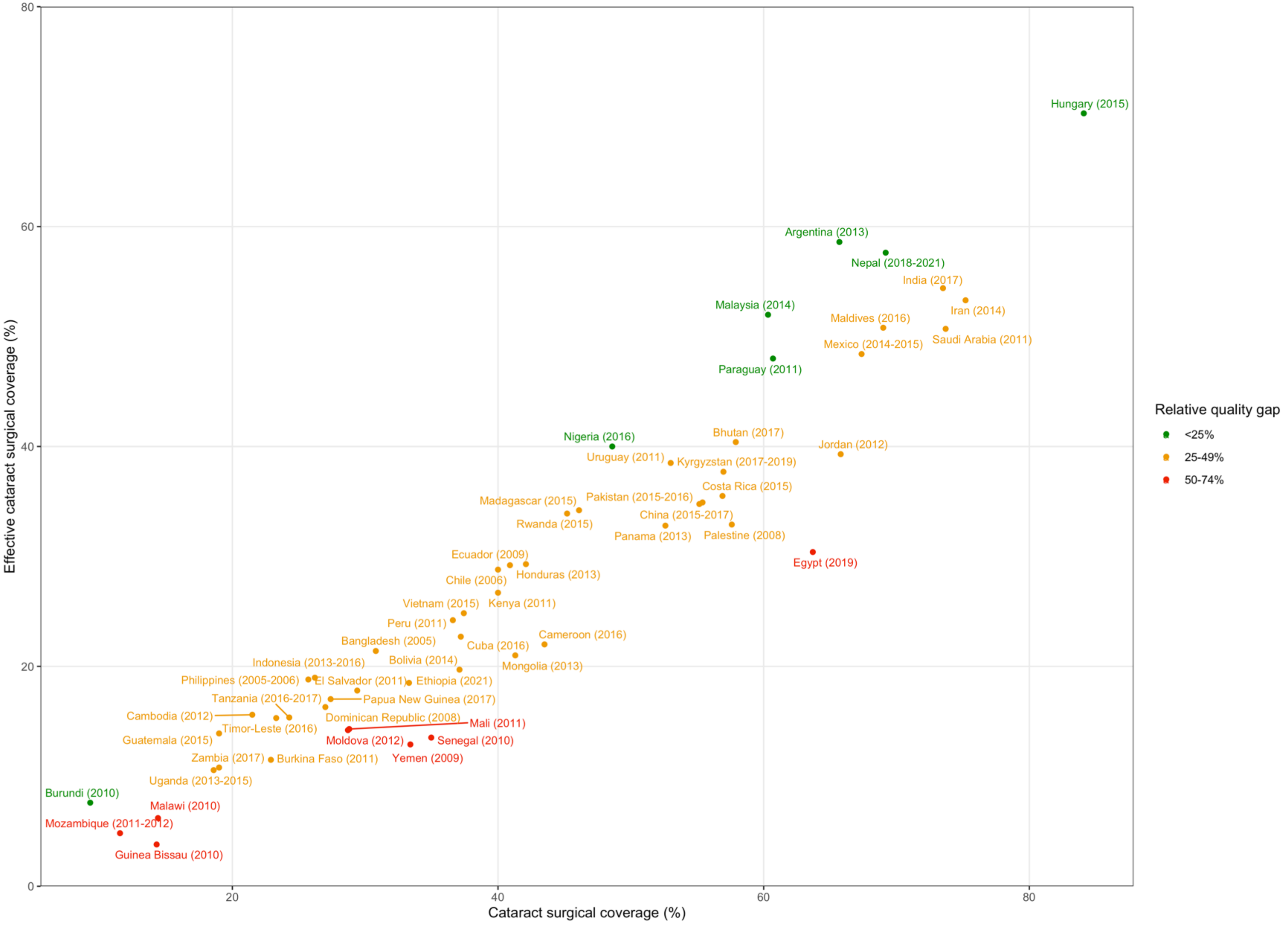

7. Forest plots of absolute (A) and relative (B) difference in male and female eCSC at the 6/18 threshold for a good outcome and operable cataract (by WHO region and overall)

(A) Absolute difference between male and female eCSC

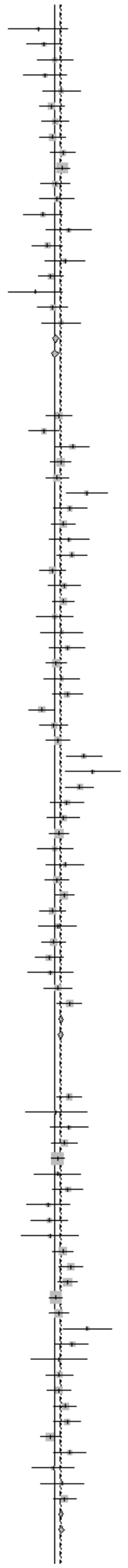

RAAB ID	Adj. male eCSC (%)	Adj. Female eCSC (%)	Risk Difference	95% CI	Weight
who_reg = Americas					
2003_Argentina_Buenos-Aires_Buenos-Aires	40.6	50.7	-10.10	[-27.55; 7.35]	0.2%
2013_Argentina	54.6	61.1	-6.50	[-17.16; 4.16]	0.5%
2014_Bolivia	19.6	19.8	-0.20	[-10.76; 10.36]	0.5%
2006_Chile_Bio-Bio	25.2	31.2	-6.00	[-18.78; 6.78]	0.4%
2015_Costa-Rica	37.6	33.9	3.70	[-7.47; 14.87]	0.5%
2016_Cuba	21.6	23.6	-2.00	[-9.41; 5.41]	0.9%
2008_Dominican-Republic	16.2	16.3	-0.10	[-7.92; 7.72]	0.8%
2009_Ecuador	28.4	30.0	-1.60	[-8.97; 5.77]	0.9%
2011_El-Salvador	20.0	15.5	4.50	[-3.09; 12.09]	0.9%
2004_Guatemala_4-states	10.3	6.1	4.20	[-0.24; 8.64]	1.5%
2015_Guatemala	13.9	13.8	0.10	[-8.40; 8.60]	0.7%
2013_Honduras	29.8	28.9	0.90	[-9.36; 11.16]	0.6%
2005_Mexico_Nuevo-Leon	27.1	34.7	-7.60	[-19.17; 3.97]	0.5%
2015_Mexico_Queretaro	46.5	38.5	8.00	[-5.27; 21.27]	0.4%
2014_Mexico_Nuevo-Leon	51.5	56.2	-4.70	[-13.86; 4.46]	0.7%
2010_Mexico_Chiapas_Central-Highland-and-Frialesca	28.5	22.7	5.80	[-6.23; 17.83]	0.4%
2013_Panama	31.5	34.1	-2.60	[-9.87; 4.67]	0.9%
2011_Paraguay	42.0	53.8	-11.80	[-27.84; 4.24]	0.3%
2011_Peru	23.4	24.7	-1.30	[-10.27; 7.67]	0.7%
2011_Uruguay	40.9	37.4	3.50	[-8.23; 15.23]	0.5%
Common effect model			0.08	[-1.90; 2.07]	--
Random effects model			-0.01	[-2.05; 2.04]	12.7%
Heterogeneity: I^2 = 0%, τ^2 = 0.8190, p = 0.58					
who_reg = South-East Asia					
2005_Bangladesh_Khulna_Satkhira	22.6	20.3	2.30	[-5.31; 9.91]	0.9%
2017_Bhutan	36.7	43.4	-6.70	[-15.84; 2.44]	0.7%
2009_Bhutan	30.6	20.3	10.30	[0.40; 20.20]	0.6%
2008_India_Uttar-Pradesh_Chitrakoot	10.3	7.1	3.20	[-3.11; 9.51]	1.1%
2017_India_Maharashtra_Pune	55.1	53.9	1.20	[-5.70; 8.10]	1.0%
2014_Indonesia_West-Java	32.5	13.9	18.60	[6.47; 30.73]	0.4%
2016_Indonesia_South-Kalimantan	20.5	11.8	8.70	[-1.18; 18.58]	0.6%
2016_Indonesia_South-Sumatra	14.5	9.9	4.60	[-2.37; 11.57]	1.0%
2013_Indonesia_South-Sulawesi	44.2	35.9	8.30	[-3.46; 20.06]	0.5%
2016_Indonesia_Maluku	22.2	12.3	9.90	[0.67; 19.13]	0.7%
2016_Indonesia_NTT	6.3	7.9	-1.60	[-9.50; 6.30]	0.8%
2015_Indonesia_Jakarta	29.6	24.3	5.30	[-4.19; 14.79]	0.6%
2015_Indonesia_East-Java	12.3	7.6	4.70	[-1.86; 11.26]	1.0%
2016_Indonesia_West-Sumatra	23.2	23.4	-0.20	[-11.09; 10.69]	0.5%
2016_Indonesia_North-Sulawesi	30.2	26.4	3.80	[-8.48; 16.08]	0.4%
2016_Indonesia_North-Sumatra	20.5	13.4	7.10	[-3.49; 17.69]	0.5%
2015_Indonesia_Central-Java	7.2	6.8	0.40	[-5.67; 6.47]	1.1%
2016_Indonesia_West-Papua	20.1	16.3	3.80	[-6.89; 14.49]	0.5%
2015_Indonesia_Bali	31.1	24.0	7.10	[-1.85; 16.05]	0.7%
2016_Maldives	47.0	55.0	-8.00	[-15.85; -0.15]	0.8%
2009_Nepal_Bheri	33.2	34.0	-0.80	[-9.01; 7.41]	0.8%
2021_Nepal_Province7	75.2	73.8	1.40	[-5.67; 8.47]	0.9%
2010_Nepal_Rapti	54.4	37.2	17.20	[6.60; 27.80]	0.5%
2008_Nepal_Karnali	54.2	31.9	22.30	[6.05; 38.55]	0.3%
2009_Nepal_Mechi	31.5	17.0	14.50	[6.01; 22.99]	0.7%
2009_Nepal_Sagarmatha	34.4	27.4	7.00	[-2.70; 16.70]	0.6%
2020_Nepal_Province4	53.7	48.6	5.10	[-4.49; 14.69]	0.6%
2018_Nepal_Province5	47.4	45.2	2.20	[-3.47; 7.87]	1.2%
2008_Nepal_Janakpur	33.0	33.1	-0.10	[-10.74; 10.54]	0.5%
2008_Nepal_Seti-and-Mahakali	48.9	43.1	5.80	[-5.36; 16.96]	0.5%
2015_Nepal_Narayani	45.6	44.5	1.10	[-5.91; 8.11]	0.9%
2020_Nepal_Province2	57.2	51.8	5.40	[-0.56; 11.36]	1.1%
2009_Nepal_Koshi	23.8	25.5	-1.70	[-9.48; 6.08]	0.8%
2010_Nepal_Dhaulagiri	39.1	37.8	1.30	[-10.08; 12.68]	0.5%
2019_Nepal_Province3	65.2	66.3	-1.10	[-8.09; 5.89]	1.0%
2019_Nepal_Karnali	50.4	54.1	-3.70	[-12.14; 4.74]	0.7%
2008_Nepal_Bagmati	65.3	68.1	-2.80	[-16.25; 10.65]	0.4%
2020_Nepal_Province1	60.0	58.4	1.60	[-6.51; 9.71]	0.8%
2016_Timor-Leste	19.9	11.5	8.40	[0.95; 15.85]	0.9%
Common effect model			3.28	[1.94; 4.62]	--
Random effects model			3.48	[1.85; 5.11]	28.2%
Heterogeneity: I^2 = 37%, τ^2 = 7.6635, p = 0.01					
who_reg = Africa					
2011_Burkina-Faso_West-Central	16.2	7.9	8.30	[0.98; 15.62]	0.9%
2010_Burundi_Ngozi-and-Kayanza	8.0	7.4	0.60	[-17.57; 18.77]	0.2%
2016_Cameroon_Yaounde	26.5	18.2	8.30	[-2.86; 19.46]	0.5%
2021_Ethiopia_Oromia_Jimma	21.8	16.5	5.30	[-2.30; 12.90]	0.9%
2010_Guinea-Bissau	4.7	3.3	1.40	[-2.36; 5.16]	1.6%
2007_Kenya_Rift-Valley_Kericho	35.2	33.9	1.30	[-12.59; 15.19]	0.3%
2011_Kenya_Coast_Kwale	31.0	23.5	7.50	[-1.38; 16.38]	0.7%
2005_Kenya_Rift-Valley_Nakuru	33.3	37.4	-4.10	[-17.03; 8.83]	0.4%
2007_Kenya_Eastern_Embu	15.6	19.3	-3.70	[-14.58; 7.18]	0.5%
2015_Madagascar_Analamanga	32.3	35.3	-3.00	[-19.87; 13.87]	0.2%
2009_Malawi_Southern-Region_Blantyre-Chikwawa-Nsan	8.9	4.3	4.60	[-1.50; 10.70]	1.1%
2011_Mali_Koulikoro	19.4	10.3	9.10	[1.93; 16.27]	0.9%
2008_Mali_Koulikoro	14.8	7.4	7.40	[1.69; 13.11]	1.2%
2011_Mozambique_Nampula	3.0	2.7	0.30	[-3.55; 4.15]	1.6%
2012_Mozambique_Sofala	8.2	6.2	2.00	[-3.73; 7.73]	1.2%
2016_Nigeria_Sokoto_Wurno	49.4	30.4	19.00	[4.50; 33.50]	0.3%
2006_Rwanda_Western	12.0	1.8	10.20	[0.63; 19.77]	0.6%
2015_Rwanda	35.6	33.2	2.40	[-14.35; 19.15]	0.3%
2010_Senegal_Kaolack	17.7	15.2	2.50	[-5.66; 10.66]	0.8%
2010_Senegal_Fatick	12.6	10.2	2.40	[-4.76; 9.56]	0.9%
2017_Tanzania_Singida	20.1	14.3	5.80	[-1.17; 12.77]	1.0%
2016_Tanzania_Morogoro	17.0	9.9	7.10	[-1.06; 15.26]	0.8%
2015_Uganda_Northern_Karamoja	7.7	10.7	-3.00	[-8.81; 2.81]	1.2%
2013_Uganda_Western_Hoima	17.3	8.6	8.70	[-1.12; 18.52]	0.6%
2011_Uganda_Western_Ntungamo	18.4	19.6	-1.20	[-13.80; 11.40]	0.4%
2012_Uganda_Central_Mubende	18.4	14.4	4.00	[-8.65; 16.65]	0.4%
2017_Zambia_Muchinga	13.7	8.0	5.70	[-1.23; 12.63]	1.0%
Common effect model			3.50	[2.07; 4.92]	--
Random effects model			3.78	[2.14; 5.43]	20.5%
Heterogeneity: I^2 = 13%, τ^2 = 2.9021, p = 0.27					

(A) Absolute difference between male and female eCSC (continued)

who_reg = Western Pacific					
2007_Cambodia	16.0	12.8	3.20	[-2.33; 8.73]	1.2%
2012_Cambodia_Takeo	16.1	15.4	0.70	[-4.32; 5.72]	1.3%
2007_China_Jiangxi_Gaoan	23.3	11.1	12.20	[1.68; 22.72]	0.5%
2007_China_Jiangxi_Wanzai	9.9	11.8	-1.90	[-13.17; 9.37]	0.5%
2010_China_Inner-Mongolia_Shangdu	22.8	7.4	15.40	[-3.49; 34.29]	0.2%
2012_China_Guangdong_Chaonan	33.7	27.1	6.60	[-4.92; 18.12]	0.5%
2008_China_Yunnan_Luliang	20.3	5.7	14.60	[5.01; 24.19]	0.6%
2012_China_Yunnan_Lancang	25.4	21.8	3.60	[-11.20; 18.40]	0.3%
2015_China_Xinjiang_Altay-and-Tacheng	32.0	35.5	-3.50	[-18.21; 11.21]	0.3%
2017_China_Sichuan_Garze	42.9	31.0	11.90	[0.24; 23.56]	0.5%
2006_China_Yunnan_Kunming	10.3	10.7	-0.40	[-7.52; 6.72]	0.9%
2012_China_Yunnan_Jianchuan	30.1	21.6	8.50	[-7.27; 24.27]	0.3%
2010_China_Hainan	32.5	22.2	10.30	[3.50; 17.10]	1.0%
2007_China_Jiangxi_Xingan	25.4	10.9	14.50	[0.08; 28.92]	0.3%
2010_China_Inner-Mongolia_Tuoketuo	3.2	16.1	-12.90	[-25.95; 0.15]	0.4%
2011_China_Sichuan_Mianning	12.1	8.1	4.00	[-2.88; 10.88]	1.0%
2011_China_Sichuan_Dechang	18.4	19.5	-1.10	[-15.51; 13.31]	0.3%
2014_Malaysia_Sarawak	40.0	35.7	4.30	[-6.90; 15.50]	0.5%
2014_Malaysia_Johor-and-Melaka	62.4	63.5	-1.10	[-14.71; 12.51]	0.4%
2014_Malaysia_Perlis-Kedah-Penang-and-Perak	58.4	63.6	-5.20	[-16.51; 6.11]	0.5%
2014_Malaysia_Kuala-Lumpur-Putrajaya-Selangor-and-	75.2	75.5	-0.30	[-10.83; 10.23]	0.5%
2014_Malaysia_Sabah-and-Labuan	32.8	29.0	3.80	[-7.89; 15.49]	0.5%
2014_Malaysia_Kelantan-Terengganu-and-Pahang	43.1	43.6	-0.50	[-13.95; 12.95]	0.4%
2013_Mongolia	23.7	19.2	4.50	[-5.57; 14.57]	0.6%
2017_Papua-New-Guinea_Highlands	8.1	5.1	3.00	[-3.24; 9.24]	1.1%
2017_Papua-New-Guinea_NCD	17.1	9.2	7.90	[-7.86; 23.66]	0.3%
2017_Papua-New-Guinea_Coastal	14.2	9.1	5.10	[-3.45; 13.65]	0.7%
2017_Papua-New-Guinea_Islands	53.6	20.7	32.90	[11.41; 54.39]	0.2%
2006_Philippines_Western-Visayas_Antique	21.8	19.6	2.20	[-9.19; 13.59]	0.5%
2005_Philippines_Western-Visayas_Negros	18.5	16.5	2.00	[-8.58; 12.58]	0.5%
2015_Vietnam_Vung-Tau	32.6	26.7	5.90	[-4.23; 16.03]	0.6%
2012_Vietnam_Nghe-An	19.4	13.9	5.50	[-1.01; 12.01]	1.0%
2011_Vietnam_Thanh-Hoa	5.5	7.1	-1.60	[-5.24; 2.04]	1.7%
2015_Vietnam_Lam-Dong	37.7	27.6	10.10	[-4.37; 24.57]	0.3%
2015_Vietnam_Ca-Mau	26.1	27.4	-1.30	[-10.71; 8.11]	0.6%
2010_Vietnam_Son-La	27.4	14.7	12.70	[-2.15; 27.55]	0.3%
2012_Vietnam_Quang-Nam	20.1	19.3	0.80	[-5.01; 6.61]	1.2%
2015_Vietnam_Tien-Giang	31.8	37.5	-5.70	[-16.47; 5.07]	0.5%
2015_Vietnam_Quang-Tri	17.3	22.2	-4.90	[-13.45; 3.65]	0.7%
2015_Vietnam_Nam-Dinh	20.6	18.5	2.10	[-7.73; 11.93]	0.6%
2015_Vietnam_Phu-Tho	19.0	19.9	-0.90	[-10.65; 8.85]	0.6%
2015_Vietnam_Ha-Tinh	19.2	20.6	-1.40	[-8.87; 6.07]	0.9%
2015_Vietnam_Binh-Duong	43.7	36.7	7.00	[-4.58; 18.58]	0.5%
2015_Vietnam_Tuyen-Quang	30.4	20.1	10.30	[-3.86; 24.46]	0.3%
2015_Vietnam_Gia-Lai	16.1	25.9	-9.80	[-21.61; 2.01]	0.5%
2015_Vietnam_Quang-Ngai	17.8	29.5	-11.70	[-20.61; -2.79]	0.7%
2015_Vietnam_Bac-Ninh	23.1	17.1	6.00	[-6.00; 18.00]	0.4%
2015_Vietnam_Dien-Bien	21.7	16.5	5.20	[-7.88; 18.28]	0.4%
Common effect model			2.10	[0.80; 3.41]	--
Random effects model			2.53	[0.85; 4.21]	28.6%
Heterogeneity: $I^2 = 37\%$, $\tau^2 = 9.5286$, $p < 0.01$					
who_reg = Eastern Mediterranean					
2019_Egypt_Sohag	35.5	26.6	8.90	[3.43; 14.37]	1.2%
2014_Iran_Kurdistan	56.6	50.0	6.60	[-2.20; 15.40]	0.7%
2009_Iran_Tehran_Varamin	61.0	57.4	3.60	[-7.01; 14.21]	0.5%
2012_Jordan_Irbid	48.5	31.9	16.60	[7.31; 25.89]	0.7%
2016_Pakistan_Khyber-Pakhtunkhwa_Swabi	26.9	20.4	6.50	[0.45; 12.55]	1.1%
2015_Pakistan_FATA_Peshawar	65.2	52.8	12.40	[1.69; 23.11]	0.5%
2016_Pakistan_Khyber-Pakhtunkhwa_Mansehra	23.2	23.0	0.20	[-7.12; 7.52]	0.9%
2008_Palestine	40.2	27.5	12.70	[4.67; 20.73]	0.8%
2011_Saudi-Arabia_Taif	59.5	43.5	16.00	[6.54; 25.46]	0.6%
2009_Yemen_Al-Amran	18.0	8.5	9.50	[2.74; 16.26]	1.0%
Common effect model			8.70	[6.28; 11.13]	--
Random effects model			8.81	[6.06; 11.56]	8.1%
Heterogeneity: $I^2 = 32\%$, $\tau^2 = 3.7733$, $p = 0.15$					
who_reg = Europe					
2015_Hungary	67.9	71.4	-3.50	[-13.50; 6.50]	0.6%
2019_Kyrgyzstan_North-east	42.0	41.2	0.80	[-14.52; 16.12]	0.3%
2017_Kyrgyzstan_Batken-OSH-and-Jalal-Abad-oblast	37.8	31.5	6.30	[-6.83; 19.43]	0.4%
2012_Moldova	15.6	12.6	3.00	[-7.38; 13.38]	0.6%
Common effect model			1.12	[-4.72; 6.95]	--
Random effects model			1.12	[-4.72; 6.95]	1.8%
Heterogeneity: $I^2 = 0\%$, $\tau^2 = 0$, $p = 0.67$					
**Common effect model**			**3.02**	**[2.33; 3.71]**	--
**Random effects model**			**3.22**	**[2.33; 4.11]**	**100.0%**

Heterogeneity: $I^2 = 36\%$, $\tau^2 = 8.8185$, $p < 0.01$
Test for subgroup differences (common effect): $\chi^2_5 = 32.40$, df = 5 ($p < 0.01$)
Test for subgroup differences (random effects): $\chi^2_5 = 27.11$, df = 5 ($p < 0.01$)

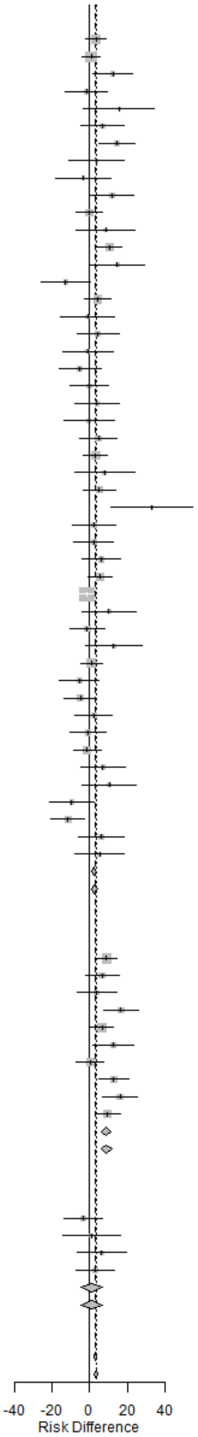

(B) Relative difference between male and female eCSC

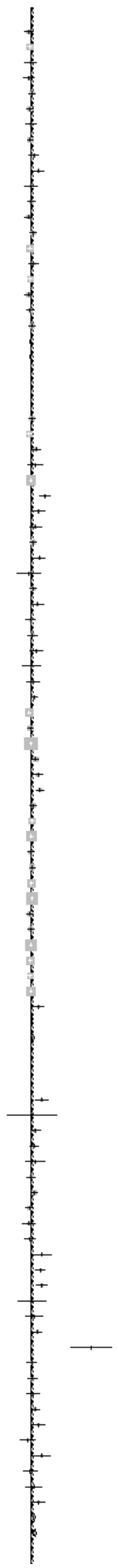

RAAB ID	Adj. male eCSC (%)	Adj. Female eCSC (%)	Relative Risk	95% CI	Weight
who_reg = Americas					
2003_Argentina_Buenos-Aires_Buenos-Aires	40.6	50.7	0.80	[0.40; 1.20]	0.7%
2013_Argentina	54.6	61.1	0.89	[0.71; 1.08]	1.1%
2014_Bolivia	19.6	19.8	0.99	[0.45; 1.53]	0.5%
2006_Chile_Bio-Bio	25.2	31.2	0.81	[0.34; 1.27]	0.6%
2015_Costa-Rica	37.6	33.9	1.11	[0.80; 1.42]	0.9%
2016_Cuba	21.6	23.6	0.92	[0.58; 1.25]	0.8%
2008_Dominican-Republic	16.2	16.3	0.99	[0.51; 1.48]	0.6%
2009_Ecuador	28.4	30.0	0.95	[0.69; 1.20]	0.9%
2011_El-Salvador	20.0	15.5	1.29	[0.86; 1.72]	0.7%
2004_Guatemala_4-states	10.3	6.1	1.69	[1.16; 2.21]	0.5%
2015_Guatemala	13.9	13.8	1.01	[0.39; 1.62]	0.5%
2013_Honduras	29.8	28.9	1.03	[0.68; 1.38]	0.8%
2005_Mexico_Nuevo-Leon	27.1	34.7	0.78	[0.40; 1.16]	0.7%
2015_Mexico_Queretaro	46.5	38.5	1.21	[0.89; 1.52]	0.8%
2014_Mexico_Nuevo-Leon	51.5	56.2	0.92	[0.74; 1.09]	1.1%
2010_Mexico_Chiapas_Central-Highland-and-Frialesca	28.5	22.7	1.26	[0.79; 1.72]	0.6%
2013_Panama	31.5	34.1	0.92	[0.70; 1.15]	1.0%
2011_Paraguay	42.0	53.8	0.78	[0.44; 1.12]	0.8%
2011_Peru	23.4	24.7	0.95	[0.57; 1.32]	0.7%
2011_Uruguay	40.9	37.4	1.09	[0.80; 1.39]	0.9%
Common effect model			0.97	[0.90; 1.04]	--
Random effects model			0.97	[0.90; 1.04]	15.3%
Heterogeneity: I^2 = 1%, τ^2 = < 0.0001, p = 0.45					
who_reg = South-East Asia					
2005_Bangladesh_Khulna_Satkhira	22.6	20.3	1.11	[0.76; 1.47]	0.8%
2017_Bhutan	36.7	43.4	0.85	[0.61; 1.08]	1.0%
2009_Bhutan	30.6	20.3	1.51	[1.11; 1.91]	0.7%
2008_India_Uttar-Pradesh_Chitrakoot	10.3	7.1	1.45	[0.75; 2.15]	0.4%
2017_India_Maharashtra_Pune	55.1	53.9	1.02	[0.90; 1.15]	1.1%
2014_Indonesia_West-Java	32.5	13.9	2.34	[1.82; 2.86]	0.5%
2016_Indonesia_South-Kalimantan	20.5	11.8	1.74	[1.14; 2.33]	0.5%
2016_Indonesia_South-Sumatra	14.5	9.9	1.46	[0.92; 2.01]	0.5%
2013_Indonesia_South-Sulawesi	44.2	35.9	1.23	[0.94; 1.52]	0.9%
2016_Indonesia_Maluku	22.2	12.3	1.80	[1.26; 2.35]	0.5%
2016_Indonesia_NTT	6.3	7.9	0.80	[-0.35; 1.94]	0.2%
2015_Indonesia_Jakarta	29.6	24.3	1.22	[0.88; 1.56]	0.8%
2015_Indonesia_East-Java	12.3	7.6	1.62	[0.98; 2.26]	0.4%
2016_Indonesia_West-Sumatra	23.2	23.4	0.99	[0.52; 1.46]	0.6%
2016_Indonesia_North-Sulawesi	30.2	26.4	1.14	[0.71; 1.57]	0.7%
2016_Indonesia_North-Sumatra	20.5	13.4	1.53	[0.93; 2.13]	0.5%
2015_Indonesia_Central-Java	7.2	6.8	1.06	[0.20; 1.92]	0.3%
2016_Indonesia_West-Papua	20.1	16.3	1.23	[0.64; 1.82]	0.5%
2015_Indonesia_Bali	31.1	24.0	1.30	[0.97; 1.62]	0.8%
2016_Maldives	47.0	55.0	0.85	[0.70; 1.01]	1.1%
2009_Nepal_Bheri	33.2	34.0	0.98	[0.73; 1.22]	1.0%
2021_Nepal_Province7	75.2	73.8	1.02	[0.92; 1.11]	1.2%
2010_Nepal_Rapti	54.4	37.2	1.46	[1.23; 1.70]	1.0%
2008_Nepal_Karnali	54.2	31.9	1.70	[1.30; 2.10]	0.7%
2009_Nepal_Mechi	31.5	17.0	1.85	[1.49; 2.21]	0.8%
2009_Nepal_Sagarmatha	34.4	27.4	1.26	[0.95; 1.57]	0.9%
2020_Nepal_Province4	53.7	48.6	1.10	[0.92; 1.29]	1.1%
2018_Nepal_Province5	47.4	45.2	1.05	[0.93; 1.17]	1.1%
2008_Nepal_Janakpur	33.0	33.1	1.00	[0.67; 1.32]	0.8%
2008_Nepal_Seti-and-Mahakali	48.9	43.1	1.13	[0.89; 1.38]	1.0%
2015_Nepal_Narayani	45.6	44.5	1.02	[0.87; 1.18]	1.1%
2020_Nepal_Province2	57.2	51.8	1.10	[0.99; 1.21]	1.2%
2009_Nepal_Koshi	23.8	25.5	0.93	[0.62; 1.25]	0.8%
2010_Nepal_Dhaulagiri	39.1	37.8	1.03	[0.74; 1.33]	0.9%
2019_Nepal_Province3	65.2	66.3	0.98	[0.88; 1.09]	1.2%
2019_Nepal_Karnali	50.4	54.1	0.93	[0.77; 1.09]	1.1%
2008_Nepal_Bagmati	65.3	68.1	0.96	[0.76; 1.16]	1.0%
2020_Nepal_Province1	60.0	58.4	1.03	[0.89; 1.16]	1.1%
2016_Timor-Leste	19.9	11.5	1.73	[1.24; 2.22]	0.6%
Common effect model			1.07	[1.04; 1.11]	--
Random effects model			1.19	[1.10; 1.28]	31.2%
Heterogeneity: I^2 = 68%, τ^2 = 0.0512, p < 0.01					
who_reg = Africa					
2011_Burkina-Faso_West-Central	16.2	7.9	2.05	[1.44; 2.66]	0.5%
2010_Burundi_Ngozi-and-Kayanza	8.0	7.4	1.08	[-1.23; 3.39]	0.0%
2016_Cameroon_Yaounde	26.5	18.2	1.46	[0.97; 1.94]	0.6%
2021_Ethiopia_Oromia_Jimma	21.8	16.5	1.32	[0.91; 1.73]	0.7%
2010_Guinea-Bissau	4.7	3.3	1.42	[0.50; 2.34]	0.3%
2007_Kenya_Rift-Valley_Kericho	35.2	33.9	1.04	[0.64; 1.44]	0.7%
2011_Kenya_Coast_Kwale	31.0	23.5	1.32	[0.99; 1.64]	0.8%
2005_Kenya_Rift-Valley_Nakuru	33.3	37.4	0.89	[0.52; 1.26]	0.8%
2007_Kenya_Eastern_Embu	15.6	19.3	0.81	[0.17; 1.45]	0.4%
2015_Madagascar_Analamanga	32.3	35.3	0.92	[0.41; 1.42]	0.6%
2009_Malawi_Southern-Region_Blantyre-Chikwawa-Nsan	8.9	4.3	2.07	[1.16; 2.98]	0.3%
2011_Mali_Koulikoro	19.4	10.3	1.88	[1.40; 2.37]	0.6%
2008_Mali_Koulikoro	14.8	7.4	2.00	[1.48; 2.52]	0.5%
2011_Mozambique_Nampula	3.0	2.7	1.11	[-0.23; 2.45]	0.1%
2012_Mozambique_Sofala	8.2	6.2	1.32	[0.54; 2.11]	0.3%
2016_Nigeria_Sokoto_Wurno	49.4	30.4	1.62	[1.25; 2.00]	0.7%
2006_Rwanda_Western	12.0	1.8	6.67	[4.72; 8.61]	0.1%
2015_Rwanda	35.6	33.2	1.07	[0.59; 1.55]	0.6%
2010_Senegal_Kaolack	17.7	15.2	1.16	[0.68; 1.65]	0.6%
2010_Senegal_Fatick	12.6	10.2	1.24	[0.62; 1.85]	0.4%
2017_Tanzania_Singida	20.1	14.3	1.41	[1.01; 1.80]	0.7%
2016_Tanzania_Morogoro	17.0	9.9	1.72	[1.09; 2.34]	0.4%
2015_Uganda_Northern_Karamoja	7.7	10.7	0.72	[0.05; 1.39]	0.4%
2013_Uganda_Western_Hoima	17.3	8.6	2.01	[1.20; 2.82]	0.3%
2011_Uganda_Western_Ntungamo	18.4	19.6	0.94	[0.27; 1.61]	0.4%
2012_Uganda_Central_Mubende	18.4	14.4	1.28	[0.52; 2.04]	0.3%
2017_Zambia_Muchinga	13.7	8.0	1.71	[1.07; 2.36]	0.4%
Common effect model			1.36	[1.25; 1.46]	--
Random effects model			1.40	[1.23; 1.56]	12.7%
Heterogeneity: I^2 = 65%, τ^2 = 0.0950, p < 0.01					

(B) Relative difference between male and female eCSC (continued)

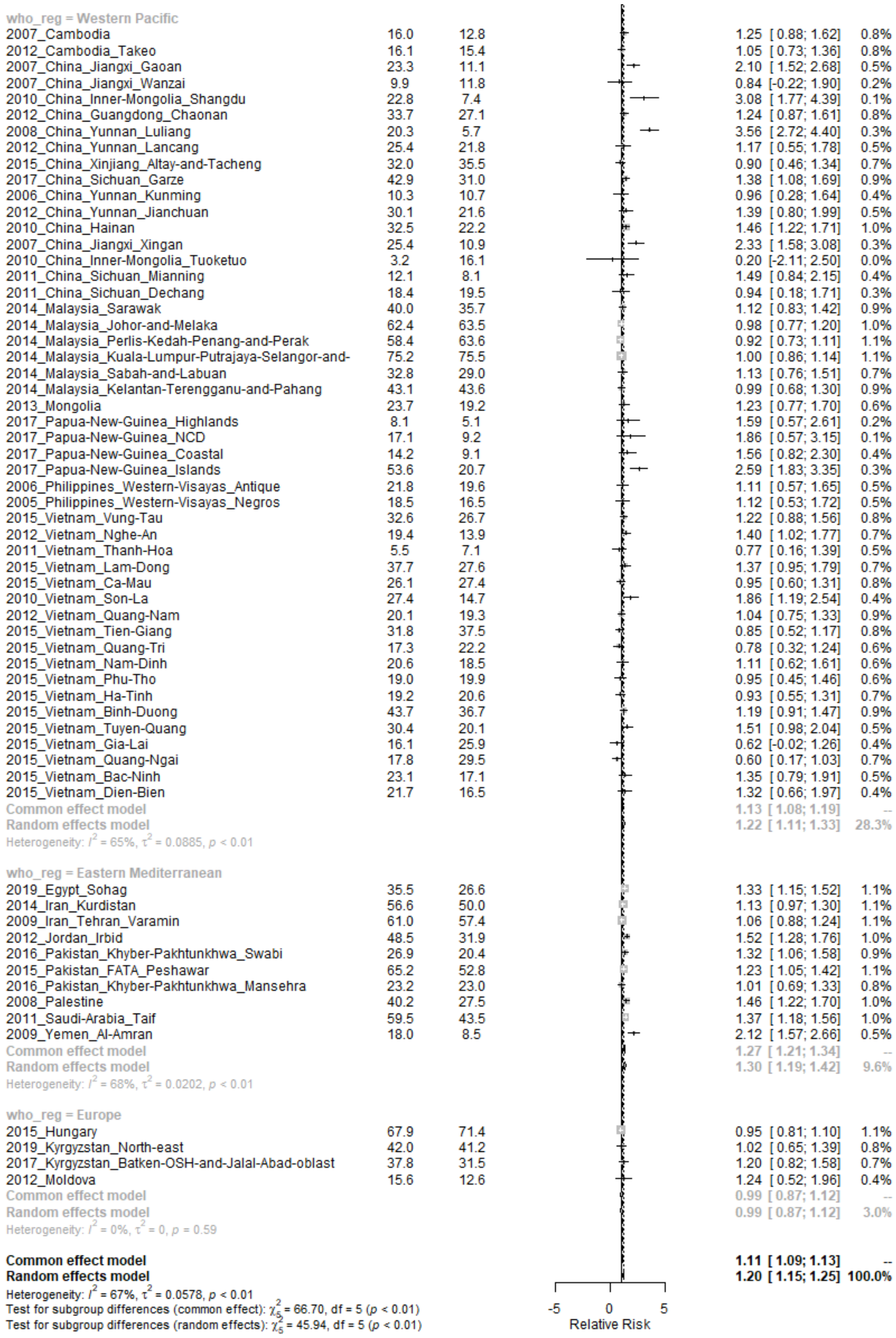

8. Forest plots of absolute (A) and relative (B) difference in male and female CSC at the 6/18 threshold for a good outcome and operable cataract (by WHO region and overall)

(A) Absolute difference between male and female CSC

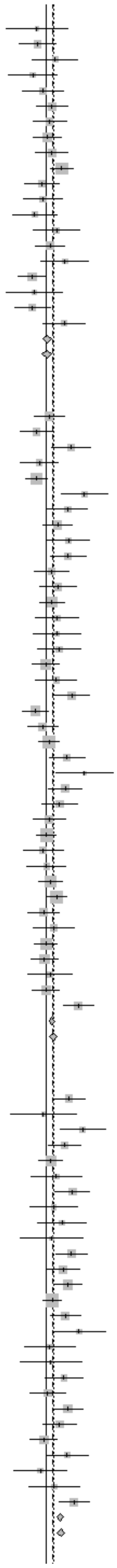

RAAB ID	Adj. male CSC (%)	Adj. Female CSC (%)	Risk Difference	95% CI	Weight
who_reg = Americas					
2003_Argentina_Buenos-Aires_Buenos-Aires	56.6	62.1	-5.50	[-22.87; 11.87]	0.3%
2013_Argentina	62.3	67.7	-5.40	[-15.72; 4.92]	0.7%
2014_Bolivia	39.8	35.2	4.60	[-8.28; 17.48]	0.5%
2006_Chile_Bio-Bio	35.2	43.1	-7.90	[-21.79; 5.99]	0.5%
2015_Costa-Rica	55.7	57.9	-2.20	[-13.73; 9.33]	0.6%
2016_Cuba	38.8	35.8	3.00	[-5.60; 11.60]	0.8%
2008_Dominican-Republic	27.8	26.2	1.60	[-7.81; 11.01]	0.8%
2009_Ecuador	41.0	40.8	0.20	[-7.77; 8.17]	0.9%
2011_El-Salvador	30.9	28.0	2.90	[-6.17; 11.97]	0.8%
2004_Guatemala_4-states	22.0	13.5	8.50	[2.37; 14.63]	1.1%
2015_Guatemala	17.3	20.0	-2.70	[-12.21; 6.81]	0.7%
2013_Honduras	40.8	43.0	-2.20	[-13.30; 8.90]	0.6%
2005_Mexico_Nuevo-Leon	42.9	49.8	-6.90	[-19.37; 5.57]	0.5%
2015_Mexico_Queretaro	64.7	59.1	5.60	[-7.46; 18.66]	0.5%
2014_Mexico_Nuevo-Leon	73.4	71.5	1.90	[-6.29; 10.09]	0.9%
2010_Mexico_Chiapas_Central-Highland-and-Frialesca	44.6	34.3	10.30	[-3.08; 23.68]	0.5%
2013_Panama	48.3	56.7	-8.40	[-16.11; -0.69]	0.9%
2011_Paraguay	57.1	64.1	-7.00	[-22.77; 8.77]	0.4%
2011_Peru	32.0	40.1	-8.10	[-18.11; 1.91]	0.7%
2011_Uruguay	59.9	50.0	9.90	[-1.93; 21.73]	0.6%
Common effect model			0.36	[-1.88; 2.59]	--
Random effects model			0.05	[-2.69; 2.78]	13.3%
Heterogeneity: I^2 = 29%, τ^2 = 10.8614, p = 0.11					
who_reg = South-East Asia					
2005_Bangladesh_Khulna_Satkhira	31.5	30.2	1.30	[-7.25; 9.85]	0.8%
2017_Bhutan	54.8	60.6	-5.80	[-15.04; 3.44]	0.8%
2009_Bhutan	43.2	29.6	13.60	[2.72; 24.48]	0.6%
2008_India_Uttar-Pradesh_Chitrakoot	46.2	50.4	-4.20	[-15.10; 6.70]	0.6%
2017_India_Maharashtra_Pune	70.3	76.0	-5.70	[-11.87; 0.47]	1.1%
2014_Indonesia_West-Java	41.8	20.6	21.20	[8.11; 34.29]	0.5%
2016_Indonesia_South-Kalimantan	29.5	17.7	11.80	[0.50; 23.10]	0.6%
2016_Indonesia_South-Sumatra	21.6	15.4	6.20	[-2.01; 14.41]	0.9%
2013_Indonesia_South-Sulawesi	53.4	41.2	12.20	[0.30; 24.10]	0.6%
2016_Indonesia_Maluku	27.8	15.7	12.10	[2.06; 22.14]	0.7%
2016_Indonesia_NTT	12.5	9.8	2.70	[-7.16; 12.56]	0.7%
2015_Indonesia_Jakarta	40.2	33.6	6.60	[-3.67; 16.87]	0.7%
2015_Indonesia_East-Java	13.5	10.6	2.90	[-4.16; 9.96]	1.0%
2016_Indonesia_West-Sumatra	36.8	31.0	5.80	[-6.44; 18.04]	0.6%
2016_Indonesia_North-Sulawesi	44.4	38.5	5.90	[-7.49; 19.29]	0.5%
2016_Indonesia_North-Sumatra	28.2	21.2	7.00	[-5.05; 19.05]	0.6%
2015_Indonesia_Central-Java	11.0	11.6	-0.60	[-8.08; 6.88]	0.9%
2016_Indonesia_West-Papua	25.8	20.4	5.40	[-6.26; 17.06]	0.6%
2015_Indonesia_Bali	47.3	33.1	14.20	[4.46; 23.94]	0.7%
2016_Maldives	66.0	72.4	-6.40	[-13.67; 0.87]	1.0%
2009_Nepal_Bheri	48.0	50.2	-2.20	[-10.89; 6.49]	0.8%
2021_Nepal_Province7	85.3	83.9	1.40	[-4.44; 7.24]	1.1%
2010_Nepal_Rapti	71.0	59.6	11.40	[1.26; 21.54]	0.7%
2008_Nepal_Karnali	66.5	45.2	21.30	[5.04; 37.56]	0.4%
2009_Nepal_Mechi	44.2	33.8	10.40	[0.76; 20.04]	0.7%
2009_Nepal_Sagarmatha	44.0	36.7	7.30	[-2.96; 17.56]	0.7%
2020_Nepal_Province4	65.9	64.5	1.40	[-7.74; 10.54]	0.8%
2018_Nepal_Province5	56.8	57.0	-0.20	[-5.83; 5.43]	1.1%
2008_Nepal_Janakpur	44.6	46.6	-2.00	[-13.26; 9.26]	0.6%
2008_Nepal_Seti-and-Mahakali	58.3	58.4	-0.10	[-11.14; 10.94]	0.6%
2015_Nepal_Narayani	64.1	61.9	2.20	[-4.59; 8.99]	1.0%
2020_Nepal_Province2	64.9	59.4	5.50	[-0.30; 11.30]	1.1%
2009_Nepal_Koshi	35.0	36.7	-1.70	[-10.37; 6.97]	0.8%
2010_Nepal_Dhaulagiri	53.6	49.6	4.00	[-7.67; 15.67]	0.6%
2019_Nepal_Province3	76.0	76.5	-0.50	[-6.77; 5.77]	1.1%
2019_Nepal_Karnali	70.2	71.5	-1.30	[-8.99; 6.39]	0.9%
2008_Nepal_Bagmati	75.1	73.1	2.00	[-10.47; 14.47]	0.5%
2020_Nepal_Province1	68.1	68.6	-0.50	[-8.18; 7.18]	0.9%
2016_Timor-Leste	33.1	15.2	17.90	[9.25; 26.55]	0.8%
Common effect model			2.96	[1.57; 4.36]	--
Random effects model			3.75	[1.71; 5.79]	29.8%
Heterogeneity: I^2 = 51%, τ^2 = 19.8804, p < 0.01					
who_reg = Africa					
2011_Burkina-Faso_West-Central	30.0	17.5	12.50	[3.07; 21.93]	0.8%
2010_Burundi_Ngozi-and-Kayanza	8.0	9.9	-1.90	[-20.67; 16.87]	0.3%
2016_Cameroon_Yaounde	54.6	34.3	20.30	[7.35; 33.25]	0.5%
2021_Ethiopia_Oromia_Jimma	39.6	29.5	10.10	[0.93; 19.27]	0.8%
2010_Guinea-Bissau	15.8	13.5	2.30	[-4.41; 9.01]	1.0%
2007_Kenya_Rift-Valley_Kericho	60.4	55.0	5.40	[-9.01; 19.81]	0.4%
2011_Kenya_Coast_Kwale	48.2	33.7	14.50	[4.78; 24.22]	0.7%
2005_Kenya_Rift-Valley_Nakuru	58.6	54.6	4.00	[-9.41; 17.41]	0.5%
2007_Kenya_Eastern_Embu	41.0	32.4	8.60	[-5.33; 22.53]	0.5%
2015_Madagascar_Analamanga	47.8	45.0	2.80	[-15.01; 20.61]	0.3%
2009_Malawi_Southern-Region_Blantyre-Chikwawa-Nsan	22.6	8.7	13.90	[5.05; 22.75]	0.8%
2011_Mali_Koulikoro	34.1	24.7	9.40	[0.33; 18.47]	0.8%
2008_Mali_Koulikoro	30.1	18.2	11.90	[4.22; 19.58]	0.9%
2011_Mozambique_Nampula	7.2	4.1	3.10	[-2.33; 8.53]	1.2%
2012_Mozambique_Sofala	23.5	13.1	10.40	[1.83; 18.97]	0.8%
2016_Nigeria_Sokoto_Wurno	57.6	39.4	18.20	[3.44; 32.96]	0.4%
2006_Rwanda_Western	18.6	16.8	1.80	[-12.30; 15.90]	0.5%
2015_Rwanda	46.8	44.4	2.40	[-15.12; 19.92]	0.3%
2010_Senegal_Kaolack	48.3	38.6	9.70	[-1.11; 20.51]	0.6%
2010_Senegal_Fatick	28.2	27.6	0.60	[-9.43; 10.63]	0.7%
2017_Tanzania_Singida	34.3	22.3	12.00	[3.73; 20.27]	0.9%
2016_Tanzania_Morogoro	24.3	17.0	7.30	[-2.37; 16.97]	0.7%
2015_Uganda_Northern_Karamoja	16.7	18.4	-1.70	[-9.47; 6.07]	0.9%
2013_Uganda_Western_Hoima	26.6	15.2	11.40	[-0.45; 23.25]	0.6%
2011_Uganda_Western_Ntungamo	29.1	32.7	-3.60	[-18.42; 11.22]	0.4%
2012_Uganda_Central_Mubende	24.0	19.8	4.20	[-9.89; 18.29]	0.5%
2017_Zambia_Muchinga	26.8	11.4	15.40	[6.73; 24.07]	0.8%
Common effect model			7.71	[5.80; 9.61]	--
Random effects model			7.94	[5.63; 10.26]	17.6%
Heterogeneity: I^2 = 25%, τ^2 = 9.4537, p = 0.12					

(A) Absolute difference between male and female CSC (continued)

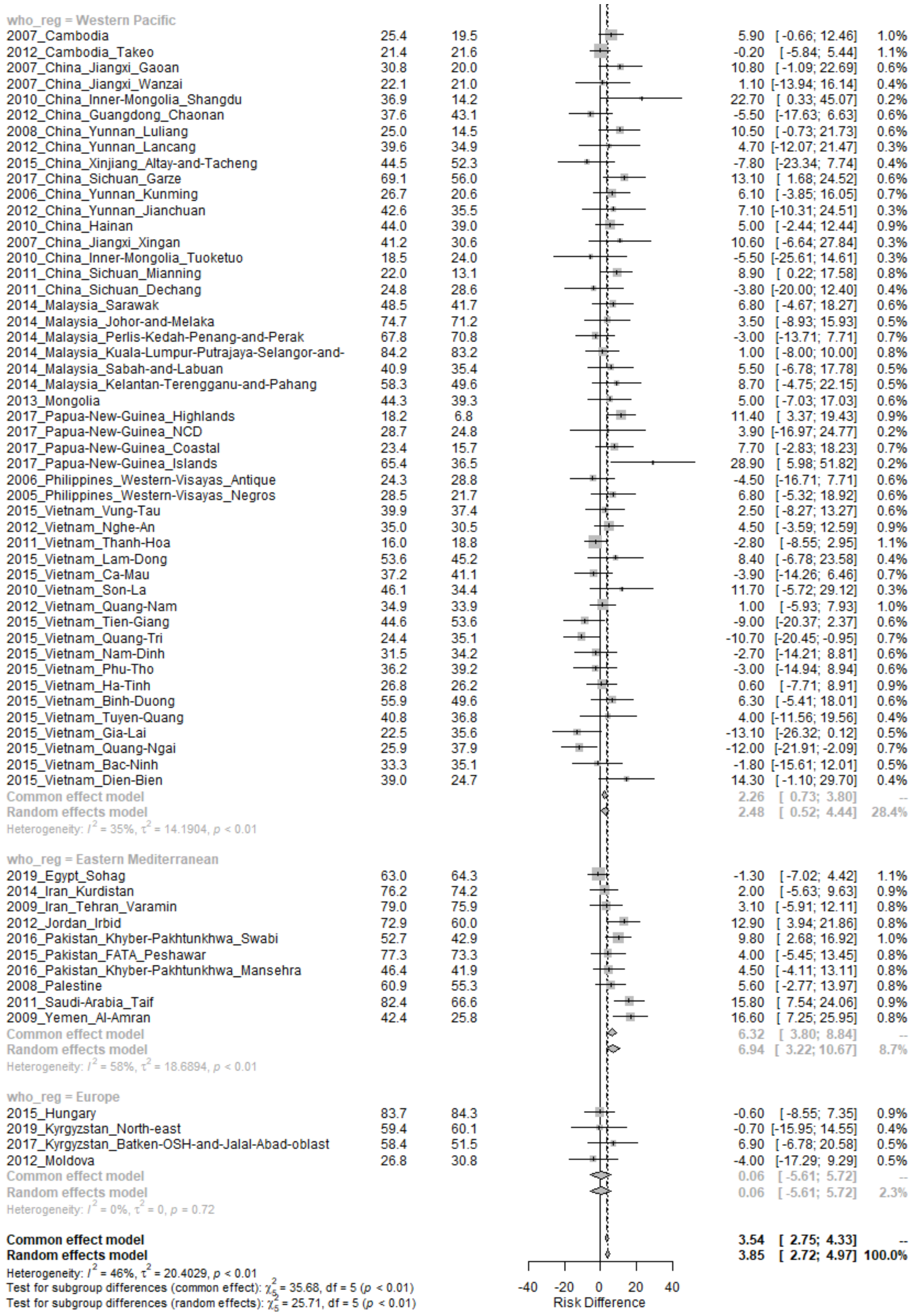

(B) Relative difference between male and female CSC

RAAB ID	Adj. male CSC (%)	Adj. Female CSC (%)	Relative Risk	95% CI	Weight
who_reg = Americas					
2003_Argentina_Buenos-Aires_Buenos-Aires	56.6	62.1	0.91	[0.61; 1.21]	0.7%
2013_Argentina	62.3	67.7	0.92	[0.76; 1.08]	0.9%
2014_Bolivia	39.8	35.2	1.13	[0.79; 1.47]	0.6%
2006_Chile_Bio-Bio	35.2	43.1	0.82	[0.45; 1.18]	0.6%
2015_Costa-Rica	55.7	57.9	0.96	[0.76; 1.17]	0.9%
2016_Cuba	38.8	35.8	1.08	[0.85; 1.31]	0.8%
2008_Dominican-Republic	27.8	26.2	1.06	[0.71; 1.41]	0.6%
2009_Ecuador	41.0	40.8	1.00	[0.81; 1.20]	0.9%
2011_El-Salvador	30.9	28.0	1.10	[0.80; 1.41]	0.7%
2004_Guatemala_4-states	22.0	13.5	1.63	[1.29; 1.97]	0.6%
2015_Guatemala	17.3	20.0	0.86	[0.34; 1.39]	0.4%
2013_Honduras	40.8	43.0	0.95	[0.68; 1.22]	0.8%
2005_Mexico_Nuevo-Leon	42.9	49.8	0.86	[0.59; 1.13]	0.7%
2015_Mexico_Queretaro	64.7	59.1	1.09	[0.88; 1.31]	0.8%
2014_Mexico_Nuevo-Leon	73.4	71.5	1.03	[0.91; 1.14]	1.0%
2010_Mexico_Chiapas_Central-Highland-and-Frialesca	44.6	34.3	1.30	[0.96; 1.64]	0.6%
2013_Panama	48.3	56.7	0.85	[0.70; 1.00]	1.0%
2011_Paraguay	57.1	64.1	0.89	[0.63; 1.15]	0.8%
2011_Peru	32.0	40.1	0.80	[0.51; 1.09]	0.7%
2011_Uruguay	59.9	50.0	1.20	[0.99; 1.41]	0.9%
Common effect model			1.00	[0.95; 1.05]	--
Random effects model			1.01	[0.94; 1.07]	15.0%
Heterogeneity: $I^2 = 42\%$, $\tau^2 = 0.0065$, $p = 0.03$					
who_reg = South-East Asia					
2005_Bangladesh_Khulna_Satkhira	31.5	30.2	1.04	[0.77; 1.32]	0.7%
2017_Bhutan	54.8	60.6	0.90	[0.74; 1.07]	0.9%
2009_Bhutan	43.2	29.6	1.46	[1.15; 1.77]	0.7%
2008_India_Uttar-Pradesh_Chitrakoot	46.2	50.4	0.92	[0.69; 1.15]	0.8%
2017_India_Maharashtra_Pune	70.3	76.0	0.92	[0.84; 1.01]	1.0%
2014_Indonesia_West-Java	41.8	20.6	2.03	[1.61; 2.45]	0.5%
2016_Indonesia_South-Kalimantan	29.5	17.7	1.67	[1.20; 2.14]	0.5%
2016_Indonesia_South-Sumatra	21.6	15.4	1.40	[0.98; 1.83]	0.5%
2013_Indonesia_South-Sulawesi	53.4	41.2	1.30	[1.05; 1.55]	0.8%
2016_Indonesia_Maluku	27.8	15.7	1.77	[1.30; 2.25]	0.4%
2016_Indonesia_NTT	12.5	9.8	1.28	[0.40; 2.15]	0.2%
2015_Indonesia_Jakarta	40.2	33.6	1.20	[0.92; 1.47]	0.7%
2015_Indonesia_East-Java	13.5	10.6	1.27	[0.70; 1.85]	0.4%
2016_Indonesia_West-Sumatra	36.8	31.0	1.19	[0.83; 1.54]	0.6%
2016_Indonesia_North-Sulawesi	44.4	38.5	1.15	[0.83; 1.47]	0.7%
2016_Indonesia_North-Sumatra	28.2	21.2	1.33	[0.86; 1.80]	0.5%
2015_Indonesia_Central-Java	11.0	11.6	0.95	[0.28; 1.62]	0.3%
2016_Indonesia_West-Papua	25.8	20.4	1.26	[0.76; 1.77]	0.4%
2015_Indonesia_Bali	47.3	33.1	1.43	[1.18; 1.67]	0.8%
2016_Maldives	66.0	72.4	0.91	[0.81; 1.02]	1.0%
2009_Nepal_Bheri	48.0	50.2	0.96	[0.78; 1.13]	0.9%
2021_Nepal_Province7	85.3	83.9	1.02	[0.95; 1.09]	1.1%
2010_Nepal_Rapti	71.0	59.6	1.19	[1.03; 1.35]	0.9%
2008_Nepal_Karnali	66.5	45.2	1.47	[1.17; 1.78]	0.7%
2009_Nepal_Mechi	44.2	33.8	1.31	[1.06; 1.56]	0.8%
2009_Nepal_Sagarmatha	44.0	36.7	1.20	[0.95; 1.45]	0.8%
2020_Nepal_Province4	65.9	64.5	1.02	[0.88; 1.16]	1.0%
2018_Nepal_Province5	56.8	57.0	1.00	[0.90; 1.10]	1.0%
2008_Nepal_Janakpur	44.6	46.6	0.96	[0.71; 1.20]	0.8%
2008_Nepal_Seti-and-Mahakali	58.3	58.4	1.00	[0.81; 1.19]	0.9%
2015_Nepal_Narayani	64.1	61.9	1.04	[0.93; 1.14]	1.0%
2020_Nepal_Province2	64.9	59.4	1.09	[1.00; 1.19]	1.0%
2009_Nepal_Koshi	35.0	36.7	0.95	[0.71; 1.20]	0.8%
2010_Nepal_Dhaulagiri	53.6	49.6	1.08	[0.85; 1.31]	0.8%
2019_Nepal_Province3	76.0	76.5	0.99	[0.91; 1.08]	1.0%
2019_Nepal_Karnali	70.2	71.5	0.98	[0.87; 1.09]	1.0%
2008_Nepal_Bagmati	75.1	73.1	1.03	[0.86; 1.20]	0.9%
2020_Nepal_Province1	68.1	68.6	0.99	[0.88; 1.11]	1.0%
2016_Timor-Leste	33.1	15.2	2.18	[1.79; 2.57]	0.6%
Common effect model			1.04	[1.01; 1.07]	--
Random effects model			1.15	[1.07; 1.23]	29.5%
Heterogeneity: $I^2 = 73\%$, $\tau^2 = 0.0429$, $p < 0.01$					
who_reg = Africa					
2011_Burkina-Faso_West-Central	30.0	17.5	1.71	[1.32; 2.11]	0.5%
2010_Burundi_Ngozi-and-Kayanza	8.0	9.9	0.81	[-1.43; 3.04]	0.0%
2016_Cameroon_Yaounde	54.6	34.3	1.59	[1.30; 1.88]	0.7%
2021_Ethiopia_Oromia_Jimma	39.6	29.5	1.34	[1.07; 1.62]	0.7%
2010_Guinea-Bissau	15.8	13.5	1.17	[0.72; 1.62]	0.5%
2007_Kenya_Rift-Valley_Kericho	60.4	55.0	1.10	[0.85; 1.35]	0.8%
2011_Kenya_Coast_Kwale	48.2	33.7	1.43	[1.19; 1.67]	0.8%
2005_Kenya_Rift-Valley_Nakuru	58.6	54.6	1.07	[0.84; 1.31]	0.8%
2007_Kenya_Eastern_Embu	41.0	32.4	1.27	[0.89; 1.64]	0.6%
2015_Madagascar_Analamanga	47.8	45.0	1.06	[0.68; 1.45]	0.6%
2009_Malawi_Southern-Region_Blantyre-Chikwawa-Nsan	22.6	8.7	2.60	[2.01; 3.18]	0.3%
2011_Mali_Koulikoro	34.1	24.7	1.38	[1.07; 1.69]	0.7%
2008_Mali_Koulikoro	30.1	18.2	1.65	[1.33; 1.98]	0.7%
2011_Mozambique_Nampula	7.2	4.1	1.76	[0.80; 2.71]	0.2%
2012_Mozambique_Sofala	23.5	13.1	1.79	[1.32; 2.27]	0.5%
2016_Nigeria_Sokoto_Wurno	57.6	39.4	1.46	[1.15; 1.78]	0.7%
2006_Rwanda_Western	18.6	16.8	1.11	[0.31; 1.90]	0.2%
2015_Rwanda	46.8	44.4	1.05	[0.67; 1.43]	0.6%
2010_Senegal_Kaolack	48.3	38.6	1.25	[1.01; 1.50]	0.8%
2010_Senegal_Fatick	28.2	27.6	1.02	[0.66; 1.38]	0.6%
2017_Tanzania_Singida	34.3	22.3	1.54	[1.25; 1.83]	0.7%
2016_Tanzania_Morogoro	24.3	17.0	1.43	[0.95; 1.90]	0.4%
2015_Uganda_Northern_Karamoja	16.7	18.4	0.91	[0.46; 1.36]	0.5%
2013_Uganda_Western_Hoima	26.6	15.2	1.75	[1.16; 2.34]	0.3%
2011_Uganda_Western_Ntungamo	29.1	32.7	0.89	[0.40; 1.38]	0.4%
2012_Uganda_Central_Mubende	24.0	19.8	1.21	[0.58; 1.85]	0.3%
2017_Zambia_Muchinga	26.8	11.4	2.35	[1.86; 2.84]	0.4%
Common effect model			1.36	[1.29; 1.43]	--
Random effects model			1.39	[1.26; 1.53]	14.3%
Heterogeneity: $I^2 = 66\%$, $\tau^2 = 0.0808$, $p < 0.01$					

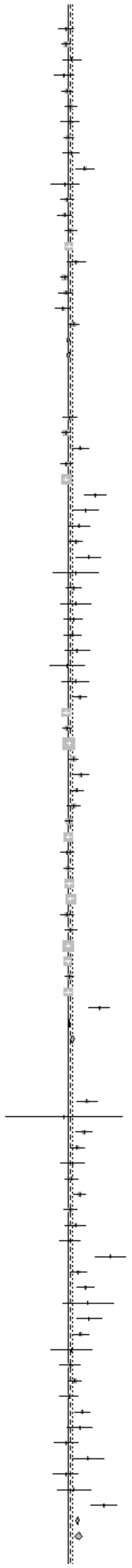

(B) Relative difference between male and female CSC (continued)

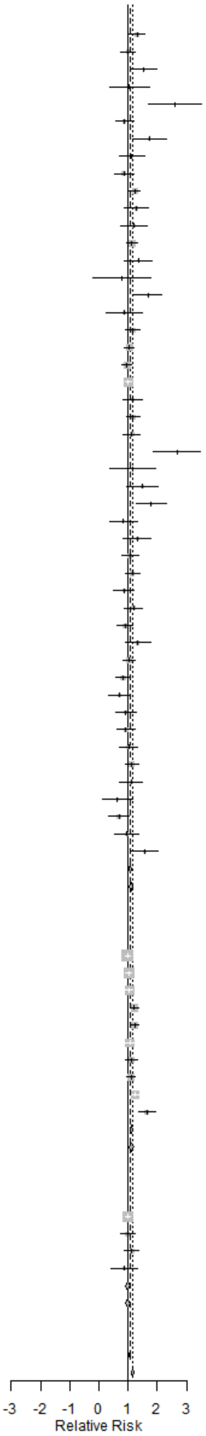

who_reg = Western Pacific				
2007_Cambodia	25.4	19.5	1.30 [1.02; 1.58]	0.7%
2012_Cambodia_Takeo	21.4	21.6	0.99 [0.73; 1.25]	0.8%
2007_China_Jiangxi_Gaoan	30.8	20.0	1.54 [1.09; 1.99]	0.5%
2007_China_Jiangxi_Wanzai	22.1	21.0	1.05 [0.36; 1.75]	0.3%
2010_China_Inner-Mongolia_Shangdu	36.9	14.2	2.60 [1.68; 3.51]	0.2%
2012_China_Guangdong_Chaonan	37.6	43.1	0.87 [0.56; 1.18]	0.7%
2008_China_Yunnan_Luliang	25.0	14.5	1.72 [1.15; 2.30]	0.4%
2012_China_Yunnan_Lancang	39.6	34.9	1.13 [0.69; 1.58]	0.5%
2015_China_Xinjiang_Altay-and-Tacheng	44.5	52.3	0.85 [0.52; 1.18]	0.6%
2017_China_Sichuan_Garze	69.1	56.0	1.23 [1.05; 1.41]	0.9%
2006_China_Yunnan_Kunming	26.7	20.6	1.30 [0.88; 1.71]	0.5%
2012_China_Yunnan_Jianchuan	42.6	35.5	1.20 [0.76; 1.64]	0.5%
2010_China_Hainan	44.0	39.0	1.13 [0.95; 1.30]	0.9%
2007_China_Jiangxi_Xingan	41.2	30.6	1.35 [0.89; 1.81]	0.5%
2010_China_Inner-Mongolia_Tuoketuo	18.5	24.0	0.77 [-0.22; 1.76]	0.2%
2011_China_Sichuan_Mianning	22.0	13.1	1.68 [1.21; 2.15]	0.5%
2011_China_Sichuan_Dechang	24.8	28.6	0.87 [0.25; 1.49]	0.3%
2014_Malaysia_Sarawak	48.5	41.7	1.16 [0.91; 1.42]	0.8%
2014_Malaysia_Johor-and-Melaka	74.7	71.2	1.05 [0.88; 1.22]	0.9%
2014_Malaysia_Perlis-Kedah-Penang-and-Perak	67.8	70.8	0.96 [0.80; 1.11]	0.9%
2014_Malaysia_Kuala-Lumpur-Putrajaya-Selangor-and-	84.2	83.2	1.01 [0.90; 1.12]	1.0%
2014_Malaysia_Sabah-and-Labuan	40.9	35.4	1.16 [0.84; 1.48]	0.7%
2014_Malaysia_Kelantan-Terengganu-and-Pahang	58.3	49.6	1.18 [0.93; 1.42]	0.8%
2013_Mongolia	44.3	39.3	1.13 [0.84; 1.41]	0.7%
2017_Papua-New-Guinea_Highlands	18.2	6.8	2.68 [1.86; 3.49]	0.2%
2017_Papua-New-Guinea_NCD	28.7	24.8	1.16 [0.37; 1.94]	0.2%
2017_Papua-New-Guinea_Coastal	23.4	15.7	1.49 [0.94; 2.04]	0.4%
2017_Papua-New-Guinea_Islands	65.4	36.5	1.79 [1.27; 2.32]	0.4%
2006_Philippines_Western-Visayas_Antique	24.3	28.8	0.84 [0.37; 1.32]	0.4%
2005_Philippines_Western-Visayas_Negros	28.5	21.7	1.31 [0.84; 1.78]	0.5%
2015_Vietnam_Vung-Tau	39.9	37.4	1.07 [0.79; 1.34]	0.7%
2012_Vietnam_Nghe-An	35.0	30.5	1.15 [0.90; 1.39]	0.8%
2011_Vietnam_Thanh-Hoa	16.0	18.8	0.85 [0.51; 1.19]	0.6%
2015_Vietnam_Lam-Dong	53.6	45.2	1.19 [0.89; 1.48]	0.7%
2015_Vietnam_Ca-Mau	37.2	41.1	0.91 [0.64; 1.17]	0.7%
2010_Vietnam_Son-La	46.1	34.4	1.34 [0.92; 1.76]	0.5%
2012_Vietnam_Quang-Nam	34.9	33.9	1.03 [0.83; 1.23]	0.9%
2015_Vietnam_Tien-Giang	44.6	53.6	0.83 [0.59; 1.08]	0.8%
2015_Vietnam_Quang-Tri	24.4	35.1	0.70 [0.34; 1.06]	0.6%
2015_Vietnam_Nam-Dinh	31.5	34.2	0.92 [0.56; 1.28]	0.6%
2015_Vietnam_Phu-Tho	36.2	39.2	0.92 [0.60; 1.25]	0.7%
2015_Vietnam_Ha-Tinh	26.8	26.2	1.02 [0.71; 1.34]	0.7%
2015_Vietnam_Binh-Duong	55.9	49.6	1.13 [0.91; 1.34]	0.8%
2015_Vietnam_Tuyen-Quang	40.8	36.8	1.11 [0.72; 1.50]	0.6%
2015_Vietnam_Gia-Lai	22.5	35.6	0.63 [0.12; 1.15]	0.4%
2015_Vietnam_Quang-Ngai	25.9	37.9	0.68 [0.34; 1.03]	0.6%
2015_Vietnam_Bac-Ninh	33.3	35.1	0.95 [0.54; 1.36]	0.5%
2015_Vietnam_Dien-Bien	39.0	24.7	1.58 [1.12; 2.04]	0.5%
Common effect model			1.08 [1.04; 1.12]	--
Random effects model			1.10 [1.04; 1.16]	28.5%
Heterogeneity: $I^2 = 53\%$, $\tau^2 = 0.0140$, $p < 0.01$				
who_reg = Eastern Mediterranean				
2019_Egypt_Sohag	63.0	64.3	0.98 [0.89; 1.07]	1.0%
2014_Iran_Kurdistan	76.2	74.2	1.03 [0.93; 1.13]	1.0%
2009_Iran_Tehran_Varamin	79.0	75.9	1.04 [0.92; 1.16]	1.0%
2012_Jordan_Irbid	72.9	60.0	1.22 [1.08; 1.35]	1.0%
2016_Pakistan_Khyber-Pakhtunkhwa_Swabi	52.7	42.9	1.23 [1.08; 1.38]	1.0%
2015_Pakistan_FATA_Peshawar	77.3	73.3	1.05 [0.93; 1.18]	1.0%
2016_Pakistan_Khyber-Pakhtunkhwa_Mansehra	46.4	41.9	1.11 [0.91; 1.30]	0.9%
2008_Palestine	60.9	55.3	1.10 [0.96; 1.25]	1.0%
2011_Saudi-Arabia_Taif	82.4	66.6	1.24 [1.12; 1.35]	1.0%
2009_Yemen_Al-Amran	42.4	25.8	1.64 [1.36; 1.93]	0.7%
Common effect model			1.10 [1.06; 1.14]	--
Random effects model			1.13 [1.05; 1.21]	9.6%
Heterogeneity: $I^2 = 74\%$, $\tau^2 = 0.0115$, $p < 0.01$				
who_reg = Europe				
2015_Hungary	83.7	84.3	0.99 [0.90; 1.09]	1.0%
2019_Kyrgyzstan_North-east	59.4	60.1	0.99 [0.73; 1.24]	0.8%
2017_Kyrgyzstan_Batken-OSH-and-Jalal-Abad-oblast	58.4	51.5	1.13 [0.89; 1.38]	0.8%
2012_Moldova	26.8	30.8	0.87 [0.40; 1.34]	0.5%
Common effect model			1.00 [0.92; 1.09]	--
Random effects model			1.00 [0.92; 1.09]	3.0%
Heterogeneity: $I^2 = 0\%$, $\tau^2 = 0$, $p = 0.70$				
**Common effect model**			**1.07 [1.05; 1.09]**	**--**
**Random effects model**			**1.15 [1.11; 1.19]**	**100.0%**

Heterogeneity: $I^2 = 69\%$, $\tau^2 = 0.0411$, $p < 0.01$
Test for subgroup differences (common effect): $\chi^2_5 = 82.22$, df = 5 ($p < 0.01$)
Test for subgroup differences (random effects): $\chi^2_5 = 32.56$, df = 5 ($p < 0.01$)
